# The mammalian sperm factor phospholipase C zeta is critical for early embryo division and pregnancy in humans and mice

**DOI:** 10.1093/humrep/deae078

**Published:** 2024-04-26

**Authors:** Junaid Kashir, Bhavesh V Mistry, Mohamed A Rajab, Lujain BuSaleh, Raed Abu-Dawud, Hala A Ahmed, Sarah Alharbi, Michail Nomikos, Saad AlHassan, Serdar Coskun, Abdullah M Assiri

**Affiliations:** Department of Biological Sciences, College of Medicine and Health Sciences, Khalifa University, Abu Dhabi, United Arab Emirates; Center for Biotechnology, Khalifa University, Abu Dhabi, United Arab Emirates; Department of Comparative Medicine, King Faisal Specialist Hospital and Research Centre, Riyadh, Saudi Arabia; Department of Comparative Medicine, King Faisal Specialist Hospital and Research Centre, Riyadh, Saudi Arabia; Department of Comparative Medicine, King Faisal Specialist Hospital and Research Centre, Riyadh, Saudi Arabia; Department of Comparative Medicine, King Faisal Specialist Hospital and Research Centre, Riyadh, Saudi Arabia; College of Medicine, Alfaisal University, Riyadh, Saudi Arabia; Department of Comparative Medicine, King Faisal Specialist Hospital and Research Centre, Riyadh, Saudi Arabia; Institute for Molecular Medicine, MSH Medical School, Hamburg, Germany; Department of Comparative Medicine, King Faisal Specialist Hospital and Research Centre, Riyadh, Saudi Arabia; Department of Pathology and Laboratory Medicine, King Faisal Specialist Hospital and Research Centre, Riyadh, Saudi Arabia; College of Medicine, QU Health, Qatar University, Doha, Qatar; Department of Obstetrics and Gynaecology, King Faisal Specialist Hospital and Research Centre, Riyadh, Saudi Arabia; College of Medicine, Alfaisal University, Riyadh, Saudi Arabia; Department of Pathology and Laboratory Medicine, King Faisal Specialist Hospital and Research Centre, Riyadh, Saudi Arabia; Department of Comparative Medicine, King Faisal Specialist Hospital and Research Centre, Riyadh, Saudi Arabia; College of Medicine, Alfaisal University, Riyadh, Saudi Arabia

**Keywords:** phospholipase C zeta (PLCzeta), oocyte activation, infertility, morphokinetics, fertilization, CRISPR, calcium, oocyte activation deficiency (OAD), animal model

## Abstract

**STUDY QUESTION:**

Are sperm phospholipase C zeta (PLCζ) profiles linked to the quality of embryogenesis and pregnancy?

**SUMMARY ANSWER:**

Sperm PLCζ levels in both mouse and humans correlate with measures of ideal embryogenesis whereby minimal levels seem to be required to result in successful pregnancy.

**WHAT IS KNOWN ALREADY:**

While causative factors underlying male infertility are multivariable, cases are increasingly associated with the efficacy of oocyte activation, which in mammals occurs in response to specific profiles of calcium (Ca^2+^) oscillations driven by sperm-specific PLCζ. Although sperm PLCζ abrogation is extensively linked with human male infertility where oocyte activation is deficient, less is clear as to whether sperm PLCζ levels or localization underlies cases of defective embryogenesis and failed pregnancy following fertility treatment.

**STUDY DESIGN, SIZE, DURATION:**

A cohort of 54 couples undergoing fertility treatment were recruited at the assisted reproductive technology laboratory at the King Faisal Hospital and Research Centre, Riyadh, Kingdom of Saudi Arabia. The recruitment criteria for males was a minimum sperm concentration of 5×10^6^ sperm/ml, while all female patients had to have at least five oocytes. Sperm PLCζ analysis was performed in research laboratories, while semen assessments were performed, and time-lapse morphokinetic data were obtained, in the fertility clinic as part of routine treatment. The CRISPR/Cas9 system was concurrently used to induce indels and single-nucleotide mutations within the *Plcζ* gene to generate strains of *Plcζ* mutant mice. Sperm PLCζ was evaluated using immunofluorescence and immunoblotting with an antibody of confirmed consistent specificity against PLCζ.

**PARTICIPANTS/MATERIALS, SETTING, METHODS:**

We evaluated PLCζ profiles in sperm samples from 54 human couples undergoing fertility treatment in the context of time-lapse morphokinetic analysis of resultant embryos, correlating such profiles to pregnancy status. Concurrently, we generated two strains of mutant *Plcζ* mice using CRISPR/Cas9, and performed IVF with wild type (WT) oocytes and using WT or mutant *Plcζ* sperm to generate embryos. We also assessed PLCζ status in WT and mutant mice sperm in the context of time-lapse morphokinetic analysis and breeding outcomes.

**MAIN RESULTS AND THE ROLE OF CHANCE:**

A significant (*P *≤* *0.05*)* positive relationship was observed between both PLCζ relative fluorescence and relative density with the times taken for both the second cell division (CC2) (*r* = 0.26 and *r* = 0.43, respectively) and the third cell division (S2) (*r* = 0.26). Examination of localization patterns also indicated significant correlations between the presence or absence of sperm PLCζ and CC2 (*r* = 0.27 and *r* = −0.27, respectively; *P *≤* *0.025). Human sperm PLCζ levels were at their highest in the ideal times of CC2 (8–12 h) compared to time ranges outside the ideal timeframe (<8 and >12 h) where levels of human sperm PLCζ were lower. Following assignment of PLCζ level thresholds, quantification revealed a significantly higher (*P *≤* *0.05) rate of successful pregnancy in values larger than the assigned cut-off for both relative fluorescence (19% vs 40%, respectively) and relative density (8% vs 54%, respectively). Immunoblotting indicated a single band for PLCζ at 74 kDa in sperm from WT mice, while a single band was also observed in sperm from heterozygous of *Plcζ* mutant mouse sperm, but at a diminished intensity. Immunofluorescent analysis indicated the previously reported (Kashir *et al.*, 2021) fluorescence patterns in WT sperm, while sperm from *Plcζ* mutant mice exhibited a significantly diminished and dispersed pattern at the acrosomal region of the sperm head. Breeding experiments indicated a significantly reduced litter size of mutant *Plcζ* male mice compared to WT mice, while IVF-generated embryos using sperm from mutant *Plcζ* mice exhibited high rates of polyspermy, and resulted in significantly reduced numbers of these embryos reaching developmental milestones.

**LIMITATIONS, REASONS FOR CAUTION:**

The human population examined was relatively small, and should be expanded to examine a larger multi-centre cohort. Infertility conditions are often multivariable, and it was not possible to evaluate all these in human patients. However, our mutant *Plcζ* mouse experiments do suggest that PLCζ plays a significant role in early embryo development.

**WIDER IMPLICATIONS OF THE FINDINGS:**

We found that minimal levels of PLCζ within a specific range were required for optimal early embryogenesis, correlating with increased pregnancy. Levels of sperm PLCζ below specific thresholds were associated with ineffective embryogenesis and lower pregnancy rates, despite eliciting successful fertilization in both mice and humans. To our knowledge, this represents the first time that PLCζ levels in sperm have been correlated to prognostic measures of embryogenic efficacy and pregnancy rates in humans. Our data suggest for the first time that the clinical utilization of PLCζ may stand to benefit not just a specific population of male infertility where oocyte activation is completely deficient (wherein PLCζ is completely defective/abrogated), but also perhaps the larger population of couples seeking fertility treatment.

**STUDY FUNDING/COMPETING INTEREST(S):**

J.K. is supported by a faculty start up grant awarded by Khalifa University (FSU-2023-015). This study was also supported by a Healthcare Research Fellowship Award (HF-14-16) from Health and Care Research Wales (HCRW) to J.K., alongside a National Science, Technology, and Innovation plan (NSTIP) project grant (15-MED4186-20) awarded by the King Abdulaziz City for Science and Technology (KACST) for J.K. and A.M.A. The authors declare no conflicts of interest.

**TRIAL REGISTRATION NUMBER:**

N/A.

## Introduction

Oocyte activation, a fundamental series of processes at fertilization that precedes embryogenesis, is underpinned by specific profiles of intracellular calcium (Ca^2+^) release in all species studied to date (Cox *et al.*, 2002; Kashir *et al.*, 2018). In mammals such as mice and humans, this manifests as a series of waves, termed Ca^2+^ oscillations, which occur in response to sperm-specific phospholipase C zeta (PLCζ). Also known as the soluble sperm factor in mammals, PLCζ hydrolyses intracellular phosphatidylinositol 4,5-bisphosphate (PIP_2_) to inositol 1,4,5-trisphosphate (IP_3_), which initiates Ca^2+^ release from the endoplasmic reticulum via IP_3_ receptors. PLCζ is present in sperm fractions capable of inducing Ca^2+^ oscillations, and immuno-depletion of PLCζ from such fractions impairs their ability to elicit Ca^2+^ release in oocytes (Fujimoto *et al.*, 2004; Kurokawa *et al.*, 2005; Kashir *et al.*, 2012b).

Sperm obtained from mice whose testicular PLCζ was disrupted by RNA interference, could only elicit abnormal Ca^2+^ oscillations and yielded reduced litter sizes (Knott *et al.*, 2005). Conversely, injection of recombinant PLCζ RNA and protein in mouse oocytes initiated Ca^2+^ release and oocyte activation similar to natural fertilization, leading to embryogenesis to the blastocyst stage (Cox *et al.*, 2002; Kouchi *et al.*, 2004), something not observed with other sperm factor candidates (Kashir *et al*., 2015). Finally, sperm from mutant mouse models resulting in PLCζ knock-out were unable to elicit Ca^2+^ release following injection into oocytes, but exhibited severely high polyspermy, reduced patterns of Ca^2+^ release, and significantly reduced litters only following IVF (Hachem *et al.*, 2017; Nozawa *et al.*, 2018). Interestingly, Hirose *et al.* (2023) showed that while assisted oocyte activation via use of Ca^2+^ ionophores could rescue fertilization, oocyte activation, and embryogenesis with the use of PLCζ knock-out sperm, this was most effectively done using PLCζ RNA. Wang *et al.* (2023) recently indicated that embryos generated by sperm from PLCζ knock-out mice also exhibited a delayed developmental profile, but also suggested a decline in the sperm and epididymal quality of such mutant mice. However, the correlation between genotype and sperm PLCζ protein localization is not clear, as all studies examining PLCζ KO thus far have not examined sperm PLCζ beyond immunoblots or have used antibodies of questionable specificity.

Sperm PLCζ has been extensively linked with cases of human male infertility where oocyte activation is deficient (OAD). Sperm from such patients are either unable to elicit Ca^2+^ release in human and mouse oocytes, or do so insufficiently even after intracytoplasmic sperm injection (injection of an individual sperm into the oocyte; ICSI) (Yoon *et al.*, 2008). PLCζ mutations have also been identified from such patients, and are predicted to modify the enzymatic structure so as to abrogate sperm PLCζ activity (Heytens *et al.*, 2009; Kashir *et al.*, 2011a,b, 2013, 2012c; Escoffier *et al.*, 2015; Ferrer-Vaquer *et al.*, 2016; Torra-Massana *et al.*, 2019). Furthermore, such cases are also intricately linked to either a complete absence or severe reduction of sperm PLCζ (Yoon *et al.*, 2008; Heytens *et al.*, 2009; Kashir *et al.*, 2010, 2013; Escoffier *et al.*, 2014; Nikiforaki *et al.*, 2014; Park *et al.*, 2015; Yelumalai *et al.*, 2015; Azad *et al.*, 2017, 2018; Tavalaee *et al.*, 2017, 2018), and are increasingly associated with multiple male-specific conditions (reviewed in Kashir (2020) and Kashir *et al.* (2022)).

In line with such research, we have also shown recently that higher levels and specific localization patterns of PLCζ in human sperm are correlated with optimal ranges of sperm fertility parameters, as cases with higher proportions of successful fertilization exhibited such high levels or more physiological patterns of PLCζ (Kashir *et al.*, 2020, 2023). Furthermore, we also demonstrated that such PLCζ parameters exhibited a negative relationship with advancing male age in mice, as the predominant pattern of PLCζ localization is altered and levels of PLCζ are generally diminished with advancing age (albeit in a strain-specific manner) (Kashir *et al.*, 2021). Collectively, such results suggest that PLCζ potentially represents a diagnostic measure not just of OAD, but also of a more general male population seeking fertility treatment. Indeed, perhaps the profiles of PLCζ may influence the efficacy of embryogenesis via profiles of elicited Ca^2+^ release (Kashir *et al.*, 2012a; Kashir, 2020), which would suggest that a much larger population may stand to benefit from relevant therapeutic or diagnostic applications.

While it is known that Ca^2+^ regulation can alter physiological processes resulting in conditions such as heart disease (Nomikos *et al*., 2014; Vassilakopoulou *et al.*, 2015), PLCζ-driven Ca^2+^ release may also underlie the efficacy of embryogenic development via the rate of cell cycle progression. Indeed, the frequency and amplitude of Ca^2+^ oscillations alters protein profiles in early embryos, embryonic compaction and blastocyst formation, as well as the rate of successful transplantation of 4-cell embryos in female mice and rabbits (Swann and Ozil, 1994; Ducibella *et al.*, 2002, 2006; Miyazaki and Ito, 2006; Ducibella and Fissore, 2008). Frequencies and amplitudes of Ca^2+^ oscillations are also directly responsible for cell cycle progression, with varying Ca^2+^ transients resulting in different rates of cell cycle progression (Ducibella *et al.*, 2002, 2006; Ducibella and Fissore, 2008). This is even more interesting when one considers that specific amounts of PLCζ delivered to human and mouse oocytes also alters the frequency and amplitude of Ca^2+^ oscillations (Yoon *et al.*, 2012; Yamaguchi *et al.*, 2017).

Fittingly, Wong *et al.* (2010) suggested initially that the time between the first and second mitosis and the time between the second and third mitosis was able to predict blastocyst formation in humans. Subsequently, correlations with embryonic morphokinetic parameters has been used as a standard practice in clinics as predictors of embryo quality, blastocyst formation and ultimately implantation, pregnancy and live birth (Cruz *et al.*, 2011; Hlinka *et al.*, 2012), with specifically the second and third round of cell divisions (within an ideal range of 8–12 h) representing the most potent indicators of embryonic developmental potential in humans (Meseguer *et al.*, 2011; Desai *et al.*, 2014; Kim *et al.*, 2017; Milewski *et al.*, 2018). Considering such potential impact of PLCζ upon embryogenesis via Ca^2+^ release profiles, as well as considering the variability of PLCζ observed amongst patients (Kashir *et al.*, 2013), it is possible that PLCζ could affect such parameters of embryogenesis (Kashir, 2020; Abdulsamad *et al.*, 2023), and perhaps also be used to predict pregnancy rates.

Herein for the first time we systematically evaluated levels and localization patterns of PLCζ in sperm from couples undergoing fertility treatment in the context of time-lapse morphokinetic analysis of the resultant embryos. We attempted to ascertain whether any specific pattern of PLCζ could be correlated with rates of cell division, parameters of embryonic quality (fragmentation, KIDScore, etc.), and ultimately the resulting pregnancy rates. Concurrently, we also utilized CRISPR/Cas9 to generate *Plcζ* mutant mice to perform similar analyses, using mutant sperm in wild type (WT) females to generate embryos in a more controlled environment given the difficulties associated with human cases in terms of variability and sample size. Such examinations potentially could stand to apply PLCζ in a larger clinical context and aid a larger number of patients seeking fertility treatment.

## Materials and methods

### Patient recruitment and sample processing

Patient samples and data for this study were obtained from consecutive treatment cycles of couples undergoing fertility treatment in the ART laboratory at the King Faisal Hospital and Research Centre (KFSH&RC), Riyadh, Kingdom of Saudi Arabia. The study was approved by the local research ethics committee and the office of research affairs at the KFSH&RC (RAC No. 2170015). Written informed consent was obtained from all couples before starting treatment. A total of 54 couples were recruited and analysed for this study. The sole recruitment criteria for males was a minimum sperm concentration of 5×10^6^ sperm/ml, while all female patients had to have at least five oocytes collected per cycle examined (i.e. couples would definitely be undergoing treatment).

Semen samples were obtained by masturbation at the clinic following 2–7 days of abstinence, and semen was allowed to liquefy for up to 60 min prior to analysis. Semen analyses (volume, concentration, and motility) were performed and evaluated following WHO recommended protocols (WHO, 2010) at the KFSH&RC ART Laboratory, which is accredited to perform semen analysis by the College of American Pathologists (CAP). Semen analysis quality control was performed by daily scrutiny of pre-recorded videos and use of Accu-Beads (Hamilton Thorne, Inc., Beverly, MA, USA). The laboratory also participates in CAP’s semen analysis external proficiency testing. Fertilization success was determined by observing second polar body extrusion and the formation of two pronuclei. Further semen and sperm processing, and density gradient washing were performed as previously described (Kashir *et al.*, 2020).

For immunofluorescent analysis, sperm were centrifuged at 500×g for 10 min and fixed by resuspending in 4% paraformaldehyde at room temperature (RT) for 15 min. Following fixation, sperm were centrifuged at 500×g for 10 min, the pellet was washed thrice using phosphate-buffered saline (PBS)+protease inhibitor cocktail (PI; complete ULTRA Tablets, Roche, USA) and the sperm pellet was resuspended in PBS+PI appropriate to the size of the pellet. Fixed sperm suspensions were spread on slides pre-coated with 0.01% poly-L-lysine (Sigma Aldrich, UK). For preparation of immunoblotting experiments, sperm concentration for each suspension was determined and appropriate volumes of sperm suspension containing 500 000 sperm/aliquot for each patient sample mixed with 5× sample loading (Laemmlli) buffer (10% (w/v) SDS; 10 mM beta-mercaptoethanol; 20% (v/v) Glycerol; 0.2M Tris–HCL, PH 6.8; 0.05% (w/v) bromophenol blue) to prepare sperm lysate aliquots. Each aliquot was briefly vortexed, snap frozen in liquid nitrogen, and stored at −80°C until required as previously described (Kashir *et al.*, 2020).

### Embryo time-lapse imaging and annotation of morphokinetic parameters

High contrast 200× images from seven focal planes for each embryo were captured by the EmbryoScope (VitroLife, Denmark) image acquisition system, capturing images every 10 min. Specific embryonic cell-cycle events, including cleavage and cell stage, blastomere symmetry, and the percent of fragmentation alongside embryo morphology, were assessed. The time for each specific cleavage event was calculated and stated as hours (h) post-insemination. Specific timepoints for each embryonic cell-cycle event were denoted as t0 for approximate time of fertilization; tPB2 for second polar body extrusion; tPNa for when the two pronuclei just start to appear; tPNf for when the two pronuclei faded; t2 for the 2-cell embryo stage; t3 for the 3-cell embryo stage; and so on until the 8-cell stage (t8). Moreover, t3-t2 (cc2), t4-t3 (s2), t5-t3 (cc3) and t8-t5 (s3) were also calculated as the duration of cell cycle intervals between two different embryonic cell-cycle stages as denoted above. Optimal ranges for kinetics were also evaluated as previously described (Wong *et al.*, 2010; Meseguer *et al.*, 2011; Kovacs, 2014; Tejera *et al.*, 2016; Milewski and Ajduk, 2017). Annotations for precise time of morphokinetic events were standardized amongst embryologists, and all embryos were assessed by a single operator. The best two embryos that showed ideal morphokinetic parameters were selected for transfer and any additional good quality embryos were frozen.

### Animal use

The mice used in this research were of C57BL/6 or CD1 strains. All procedures and experiments involving laboratory animals were reviewed and approved by the Institutional Animal Care and Use Committees (IACUC) and Research Advisory Council (RAC) (Project RAC No. 2160014) at the KFSHR&C. All mice were raised and maintained in the Association for Assessment and Accreditation of Laboratory Animal Care International accredited laboratory animal facility at the KFSHR&C, Riyadh, Saudi Arabia, under controlled conditions of 22–24°C temperature, 12:12 h light/dark cycle and 50–60% humidity with *ad libitum* access to standard chow feed and water. All the experimental procedures involving mice were carried out according to the ARRIVE (Animal Research: Reporting of In Vivo Experiments) and IACUC guidelines.

### Generation of *plcζ* mutant mice by CRISPR/Cas9-based genome editing

The CRISPR/Cas9 system was used to induce indels or single-nucleotide mutations within the *Plcζ* gene to generate strains of *Plcζ* mutant mice using C57BL/6 strains as the genomic background, as described previously with minor modifications (Harms *et al.*, 2014; Yang *et al.*, 2014). Candidate single-guide RNAs (sgRNAs) targeting the sequences of exon 3 or exon 6 sequences of mouse *Plcζ*, were identified and translated to crRNA, using the web tool CRISPOR (Concordet and Haeussler, 2018). The single-strand oligodeoxynucleotide (ssODN) donor sequence, which constitutes the DNA repair template containing the desired nucleotide modification specific to the exon 6 sequence of mouse *Plcζ*, was designed such that the desired mutation was introduced, while the PAM sequence was abolished to prevent potential re-targeting by the Cas9 protein. The ssODN, the crRNAs, and the tracRNA (Supplementary Table S1) were purchased from Integrated DNA Technologies (IDT; CA, USA).

*Plcζ* mutagenesis was performed as previously described (Pu *et al.*, 2019). Pronuclear microinjection was used to deliver the CRISPR/Cas9 reagent mixture into mouse zygotes using an Eppendorf Cell Tram microinjection system (Eppendorf AG, Hamburg, Germany) mounted on a Nikon ECLIPSE Ti-U inverted microscope (Nikon Corporation, Tokyo, Japan). Briefly, mouse zygotes were obtained by mating superovulated female C57BL/6 (3–4 weeks old) mice with proven stud C57BL/6 male mice and collecting zygotes the next day from oviducts as described (Henao-Mejia *et al.*, 2016). Healthy zygotes with good morphology and clearly visible two pronuclei were selected for microinjection. To generate *Plcζ* indels driven by the non-homologous end joining DNA repair pathway in the exon 3 (E3) region of mouse *Plcζ*, the CRISPR/Cas9 reagent mixture containing a final concentration of 50 ng/µl of crRNA specific to exon 3 sequence, 100 ng/µl of tracrRNA and 100 ng/µl of Cas9 nuclease protein (NEB; cat no. M0386M) was prepared in injection buffer (8 mM Tris–HCl, 0.15 mM EDTA, pH 7.4).

To generate the N241L substitution in exon 6 (E6) of *Plcζ* driven by the homology-direct repair pathway which resulted in a tandem insertion of the corresponding crRNA, the CRISPR/Cas9 reagent mixture containing 50 ng/µl of crRNA specific to exon 6 sequence, 100 ng/µl of tracrRNA, 100 ng/µl of ssODN containing the desired nucleotide modification, and 100 ng/µl of Cas9 nuclease protein (NEB; cat no. M0386M) was prepared in injection buffer (8 mM Tris–HCl, 0.15 mM EDTA, pH 7.4).

Healthy zygotes were transferred to microinjection dishes containing HEPES-buffered medium under mineral oil. For each of the CRISPR/Cas9 reagent mixtures described above, 3pl were separately microinjected into the pronucleus of around 150–200 zygotes in two independent sessions to induce mutagenesis of *Plcζ* E3 or E6 sequences. Injected zygotes were transferred to potassium simplex optimized medium (KSOM) with amino acids and incubated at 37°C in 5% CO_2_ and 95% humidity for about 1 h. Around 25–30 surviving zygotes were surgically implanted into the oviducts of 0.5 days post-coitum pseudopregnant CD1 recipient female mice and viable offspring were obtained. All mice showed normal development and appeared healthy. The resulting offspring were analysed for editing of the *Plcζ* gene.

Positive founder (F0) mice for *Plcζ* mutations were backcrossed with WT mice to produce the F1 generation of heterozygous mutant mice. F1 heterozygous *Plcζ* mutant offspring were further subjected to six rounds of backcrossing to eliminate potential off-target mutations. Following backcrossing, the top five predicted off-target sites for each crRNA predicted using CRISPOR (Concordet and Haeussler, 2018), were assessed for both E3 and E6 by sequencing of PCR products. Heterozygous mice not carrying off-target mutations were then subjected to breeding with heterozygous male/female counterparts to generate homozygous E3 or E6 *Plcζ* mutant mice. Colonies were maintained by homozygous and/or heterozygous males and females mating, with each litter genotyped by PCR.

### Genotyping of mutant founders and their offspring

Genomic DNA (gDNA) was extracted from tail biopsies of founder mice and their progenies using the Gentra Puregene Mouse Tail Kit (Qiagen, Hilden, Germany), following the manufacturer’s protocol. For genotyping, the genomic sequences around the crRNA target sites for *Plcζ* E3 and E6 mutagenesis were amplified using PCR reactions, containing 3 µl gDNA (30 ng) and 1 µl of appropriate target specific forward and reverse primers (10 µM each; Supplementary Table S2), prepared in a total volume of 25 µl HotStarTaq DNA Polymerase PCR reaction mixture according to the manufacturer’s instructions (Qiagen, Hilden, Germany). PCR products were purified using the QIAQuick PCR purification kit (Qiagen, Hilden, Germany) according to the manufacturer’s instructions and subject to Sanger sequencing. The sequences were analysed and compared to the WT *Plcζ* sequence using DNASTAR sequence analysis software package (DNASTAR, Inc., WI, USA). Once the *Plcζ* exon 3 and exon 6 mutant mice strains were established, conventional PCR protocols were developed for each mutation by designing mutant sequence specific primers for routine genotyping of mutant mice colonies.

### Off-target analysis

Putative off-target sites for *Plcζ* E3 or E6 crRNA were predicted by the CRISPOR design tool (Concordet and Haeussler, 2018). Mutant mice were back-crossed over six generations to breed out potential off-target effects. The top five putative off-target sites were selected for each crRNA target sequence and primers specific to putative off-target sequences (Supplementary Table S3) were designed to amplify the potential off-target sites from the gDNA of *Plcζ* mutant mice using HotStarTaq DNA Polymerase PCR reaction kit according to the manufacturer’s instructions (Qiagen, Hilden, Germany). The PCR products were purified using the QIAQuick PCR purification kit (Qiagen, Hilden, Germany) according to the manufacturer’s instructions and sent for Sanger sequencing at the KFSH&RC sequencing facility. The potential off-target sequences were analysed and lack of mutations within potential off-target sequences were confirmed using DNASTAR sequence analysis software package (DNASTAR, Inc., WI, USA).

### Mating behaviour and fertility test

Sexually mature male mice (n = 3) of each genotype (WT, E3^−/−^, and E6^−/−^) were each mated individually with three sexually mature (8–10 weeks old) WT female mice in a controlled breeding experiment. Females were observed for the presence of vaginal plugs and signs of pregnancy. Litter numbers and pups produced by controlled breeding experiments for each genotype of mice were recorded.

### Mouse tissue histology

Histological analyses were performed on testes and epididymides from a representative pool of WT and *Plcζ* mutant mice (experiments performed on ×3 biological repeats) to evaluate spermatogenesis and morphological integrity of tissue. Collected tissue were submerged in 4% paraformaldehyde (epididymi) or in Bouin’s fixative (testes) for overnight fixation at 4°C. Fixed tissue were washed with distilled water and dehydrated in a series of 70–100% ethanol solutions, followed by paraffin-embedding. Tissue sections of 5 µm thickness were prepared on glass slides using a microtome (Leica RM2155, Leica Biosystems, Buffalo Grove, IL, USA). Paraffin embedded sections were deparaffinized, rehydrated and stained with hematoxylin and eosin or periodic acid-Schiff (PAS) staining following standard protocols. Stained testis and epididymis sections were observed at room temperature using Olympus BX53 (Olympus, Center Valley, PA, USA) optical microscope. Digital images were captured using OLYMPUS DP72 microscope camera (Olympus, Center Valley, PA, USA) and OLYMPUS cellSens Entry 1.6 imaging software (Olympus, Center Valley, PA, USA). No post-acquisition modifications were made to the original images.

### Mouse sperm extraction and preparation

Sperm from WT and mutant mice were isolated from cauda epididymides as previously described (Kashir *et al.*, 2021). Briefly, cauda epididymis from 8 to 12 weeks old mice of each genotype (n = 3/genotype) were dissected and collected in pre-warmed 1 ml of M2 medium (Sigma Aldrich, UK) in 35 mm culture dishes at 37°C. Sperm were allowed to swim out by making multiple incisions on the cauda epididymides and incubating them for 15–30 min at 37°C under 5% CO_2_. Cauda tissue was discarded, and the sperm suspension was centrifuged at 500 g for 10 min at RT. The resulting sperm pellet was washed by re-suspending it in pre-warmed PBS+PI (complete ULTRA Tablets, Roche, USA), and centrifuging at 500×g for 10 min at RT. Finally, the sperm pellet was resuspended in appropriate volumes of PBS+PI. Sperm counts were performed using a hemocytometer chamber (Hausser Scientific, USA), and single-use aliquots consisting of 1 million sperm per aliquot in appropriate volumes of 2× reducing Laemelli sample loading buffer (BIO-RAD, USA) were used for immunoblotting experiments. Aliquots were snap-frozen in liquid nitrogen and stored at −80°C until required. The remaining sperm suspension was centrifuged at 500×g for 10 min at RT, and the sperm pellet resuspended in 4% paraformaldehyde, and fixed at RT for 15 min. Sperm were washed again twice with PBS and resuspended in 0.2 M sucrose (Sigma Aldrich, UK) with PI. Sperm were then smeared onto slides pre-coated with 0.01% poly-L-lysine (Sigma Aldrich, UK), and stored for later use.

### IVF and time lapse imaging of mutant mice

Mouse IVF was performed using the Center for Animal Resources and Development (CARD) medium kit (Cosmo Bio, USA) developed by the CARD, Kumamoto University, Japan, following the manufacturer’s recommended protocol. All utilized media were pre-warmed and calibrated prior to experiments at 37°C under 5% CO_2_ in air. Briefly, WT females (21–25 days) were subject to superovulation by injecting 7.5 IU of pregnant mare’s serum gonadotropin (PMSG; Sigma Aldrich, UK) i.p., followed by injection of 7.5 IU i.p. of human chorionic gonadotropin (hCG; Sigma Aldrich, UK) 48–52 h later. Female mice were euthanized by cervical dislocation ∼12 h after hCG injection, and cumulous oocyte complexes (COCs) were isolated from oviducts in CARD medium drops submerged in mineral oil.

WT and mutant sperm were isolated from cauda epididymi and pre-incubated as per the recommended protocol (CARD medium kit; Cosmo Bio, USA). Following recommended incubation, sperm were added to COC drops and incubated (37°C, 5% CO_2_ in air) for ∼3–4 h to allow for fertilization to occur. COCs were then subjected to serial washing in drops of modified human tubal fluid (Sigma Aldrich, UK), and subjected to a further incubation of ∼4–6 h. In this time cumulus cells detached from the zygotes which were further washed in drops of KSOM media, with any degenerating zygotes being discarded. Viable zygotes were transferred to drops of KSOM media overlayed with mineral oil in EGPS-010 Embryo GPS^®^ dishes (SunIVF, USA) for time-lapse imaging. Mouse embryo time-lapse imaging was performed using a CCM-IVF embryo observation system (Astec-Bio, Japan), which captured single focal plane images at 10× magnification for each set of embryos, capturing images every 10 min for 5 days. Image annotation and developmental milestones were recorded as previously described for human embryos. Experiments were performed in triplicate using WT, E3 mutant and E6 mutant sperm, and were repeated using three separate animals for each strain (3×3 repeats).

### Immunofluorescence microscopy

Immunofluorescence processing and imaging was performed as previously described (Kashir *et al.*, 2013, 2016, 2020). Fixed sperm were added to slides previously coated with 0.01% (w/v) poly-L-lysine solution (Sigma Aldrich, UK), within hydrophobic moulds drawn using a PAP pen (Vector laboratories). Sperm were permeabilized with PBS-1% Triton X-100 (v/v) for 1 h at RT, and blocked by PBS-10% bovine serum albumin (BSA; Sigma Aldrich, UK). Primary antibodies were added at appropriate dilutions with PBS-5% BSA overnight at 4°C, following which AlexaFluor-488 conjugated goat anti-rabbit secondary antibody (1:100; Life Technologies, UK; Catalog number: A48282), diluted in PBS-5% BSA was added for 1 h at RT. Washing with PBS were performed between all steps. Sperm slides treated with only secondary antibody dilutions only were used as negative controls for immunofluorescence imaging. Slides were mounted using Vectashield mounting medium containing 4′-6-diamidino-2-phenylindole (Vector Laboratories, USA). The antibodies used were the EF polyclonal antibody (EF pAb; raised in rabbits against a 16-mer-human PLCζ peptide sequence—8SKIQDDFRGGKINLEK23) (Nomikos *et al.*, 2013, 2014, 2015a,b, 2017; Kashir *et al.*, 2016, 2020) and the I33 monoclonal antibody (I33 mAb; raised by injecting a recombinant PLCζ protein fragment corresponding to position 155–465 of the human PLCζ amino acid sequence; UniProt ID: Q86YW0; into rabbits, followed by cell fusion into rabbit hybridoma cells and affinity purification) (Kashir *et al.*, 2020).

Images were captured at ×40 and ×100 magnifications (using oil-immersion, type FF, Electron Microscopy Sciences, Cat. No. 16916-04), using an OLYMPUS BX53 fluorescence microscope (Olympus, USA) as previously described by Kashir *et al.* (2020). An OLYMPUS DP73 camera (Olympus, USA) was used to capture images using OLYMPUS CellSens Entry software (Olympus, USA). Brightfield images were captured alongside the corresponding fluorescence images obtained using a fluorescein isothiocyanate filer. All images were captured at the same exposure time throughout patients and samples (100 cells per patient/sample) (Kashir *et al.*, 2020).

### Immunoblotting and antibody validation

As previously described (Kashir *et al.*, 2020), pre-prepared single-use sperm lysate aliquots (5×10^5^ human sperm/aliquot and 1×10^6^ mouse sperm/aliquot) were thawed and heated at 101°C for 5 min, vortexed, cooled on ice for 5 min, and briefly centrifuged before loading onto gels. Sperm protein samples (500 000 sperm/lane) were separated through SDS–PAGE using 10% gels and transferred onto polyvinylidene difluoride membranes (Amersham Hybond, GE Healthcare Life Sciences, USA), using wet-transfer at 100 V for 1 h. Membranes were stained with 0.1% (w/v) ponceau stain prepared in 5% (v/v) acetic acid solution to assess transfer and protein separation efficacy, after which the stain was washed with PBS-T. Membranes were incubated overnight at 4°C with the primary EF pAb or I33 mAb (diluted 1:1000), followed by incubation with an anti-rabbit secondary antibody conjugated with horseradish peroxidase (HRP) for 1 h at RT diluted at 1:1000. HRP detection was achieved using the ECL select chemiluminescence kit (GE Life Sciences, UK), following the manufacturer’s recommended protocol (GE Life Sciences, UK). Chemiluminescence was detected using the ImageQuant LAS4000 (GE Healthcare Life Sciences, USA) image system (Kashir *et al.*, 2020).

### Sperm PLCζ analysis

As previously described (Kashir *et al.*, 2013, 2016, 2020), sperm PLCζ fluorescence quantification and statistical analysis was performed on images obtained at 40× magnification using the ImageJ software package (National Institutes of Health, USA) with the regions of interest tool, which were also used to assess localization patterns. For each patient, 100 cells were examined, and total fluorescence subtracted by background fluorescence was used to yield relative fluorescence. Statistical analyses were performed using Prism 7.0 (Graphpad, USA) as previously described (Benjamini *et al.*, 2006; Kashir *et al.*, 2013, 2016, 2020). A *P*-value ≤0.05 was considered statistically significant.

### Statistical analysis

Differences between two variables were examined using the *t*-test with Welch’s correction for unequal standard deviations. Multi-variable examinations were performed (including multiple comparisons tests) using one-way or two-way ANOVA as appropriate followed by Tukey *post hoc* analysis. Pearson correlation coefficients were also calculated for all variables. Data represented as proportions (%) were ARCSIN transformed prior to statistical analysis to avoid truncation of data. Significance levels were adjusted for localization pattern correlations, where the *post hoc* corrected significance value was adjusted according to the number of localization patterns observed (0.05/n, where n is the number of distinct patterns observed). For localization pattern analyses to be considered significant, a *P*-value ≤0.0125 was considered statistically significant. Significance between nominal variables (namely pregnancy and no pregnancy) were measured using the Pearson chi-square (χ^2^) test.

## Results

### Human sperm PLCζ correlations with time-lapse imaging parameters of embryos

**Figure 1.**
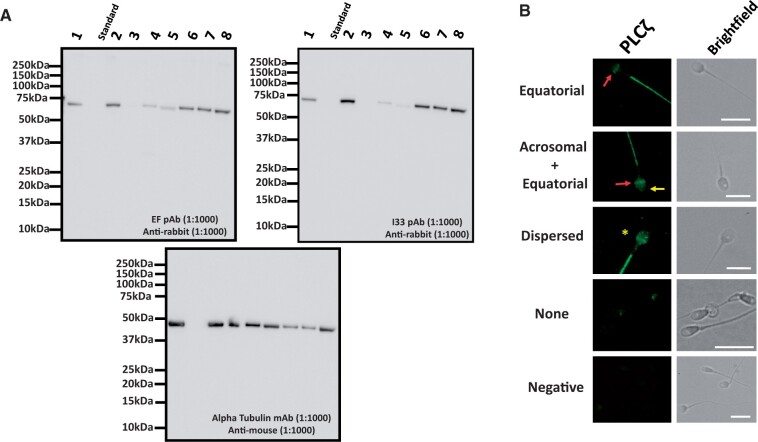
**Representative images indicating analysis of PLCζ in sperm from various patients.** (**A**) Immunoblotting using both the EF pAb (left panel) and the I33 mAB (right panel) recognized a single band at the expected molecular weight for human sperm PLCζ (∼70kDa) at varying intensities depending upon patient examined. Images are representative of at least three repeats throughout our study population. (**B**) Representative images of PLCζ (green fluorescence; left panel) alongside corresponding brightfield images (right panel) in human sperm from patients undergoing fertility treatment. Patterns observed included equatorial (red arrows); acrosomal (yellow arrow)+equatorial; dispersed (yellow star) and none (no PLCζ fluorescence observed). Bottom-most panels indicate negative controls where sperm were treated without primary antibody. Representative images obtained were captured at 100×, while white scale bars represent 10 μm. Images are representative of 100 cells examined for each group. pAB: polyclonal antibody; mAB: monoclonal antibody.

Of the 54 couples recruited for this study, the average female age was 33.3 ± 4.7 years, while the average male age was 28 ± 6.4 years. The average duration of infertility for couples was 4.4 ± 3.2 years. Immunoblotting of PLCζ in human sperm yielded a single band at ∼70 kDa of varying intensity between patients (Fig. 1A) and immunofluorescence revealed localization patterns (equatorial, acrosomal+equatorial, dispersed; and no localization, Fig. 1B) as previously described (Kashir *et al.*, 2016, 2020, 2023). These profiles were compared with parameters of sperm health and male/female age (Supplementary Table S4), with only motility exhibiting a significant negative correlation with male age (*r* = −0.3; *P *<* *0.05; Supplementary Table S5). We also observed a significantly higher level of PLCζ in sperm from cases exhibiting successful fertilization (Supplementary Fig. S1) in line with previous reports (Kashir *et al.*, 2020, 2023). Interestingly, while younger couples were more successful in achieving pregnancy, fertilization rates did not significantly differ overall between couples who did or did not achieve pregnancy (Supplementary Fig. S2).

A significant (*P *≤* *0.05) positive relationship was observed for sperm PLCζ levels (both relative fluorescence and density) with the time for the respective embryos to undergo the second cell division (CC2; *r* = 0.26 and *r* = 0.43, respectively), and there was also a positive relationship between sperm PLCζ relative fluorescence and the time for the respective embryos to undergo the third cell division (S2; *r* = 0.26). Relative density of sperm PLCζ also exhibited a significant negative relationship between KIDScore ≤ 4 (*r* = −0.45) but showed a positive relationship with KIDScore = 5 (*r* = 0.45) (Table 1) of the respective embryos. Other observed parameters did not exhibit significant relationships (*P *>* *0.05). Examination of localization patterns also indicated significant correlations between the overall presence or absence of sperm PLCζ and the CC2 time of embryos (*r* = 0.27 and *r* = −0.27, respectively; *P *≤* *0.025), although no further significant correlations were observed with regards to specific localization patterns or with any other embryo parameter examined (Table 2).

**Table 1.** Correlative analysis between morphokinetic milestones of respective embryos and PLCζ quantification in sperm.

Quantification method	tPNa	tPNf	CC2	S2	t2	t3	t4	t5	t6	t7	t8	KIDScore ≤ 4	KIDScore = 5
**Relative fluorescence (a.u.)**	n.s.	n.s.	***r* = 0.23 (*P < *0.05)**	**0.26 (*P < *0.05)**	n.s.	n.s.	n.s.	n.s.	n.s.	n.s.	n.s.	n.s.	n.s.
**Relative density (a.u.)**	n.s.	n.s.	***r* = 0.43 (*P < *0.05)**	n.s.	n.s.	n.s.	n.s.	n.s.	n.s.	n.s.	n.s.	***r*** **=** −**0.45 (*P < *0.05)**	***r* = 0.45 (*P < *0.05)**

PLCζ was quantified in sperm from corresponding human patients undergoing fertility treatment using relative fluorescence and relative density. Statistically significant (*P *≤* *0.05) differences are indicated, along with the corresponding Pearson’s correlation coefficient (*r*; positive values indicate a positive correlation). n.s., non-significant differences; a.u., arbitrary units; tPNa, time to pronuclear appearance; tPNf, time to pronuclear fading; CC2, time taken between the second and third cell cycle; S2, time taken between the third and fourth cell cycle; t2–t8, time taken to get to number of cells (2–8).

**Table 2.** Correlative analysis between morphokinetic milestones of respective embryos and proportions of sperm with specific PLCζ localization patterns.

PLCζ localization	tPNa	tPNf	CC2	S2	t2	t3	t4	t5	t6	t7	t8	KIDScore ≤ 4	KIDScore = 5
**Total with PLCζ (regardless of pattern; %)**	n.s.	n.s.	***r* = 0.27 (*P < *0.025)**	n.s.	n.s.	n.s.	n.s.	n.s.	n.s.	n.s.	n.s.	n.s.	n.s.
**Total without PLCζ (regardless of pattern; %)**	n.s.	n.s.	***r*** **=** −**0.27 (*P < *0.025)**	n.s.	n.s.	n.s.	n.s.	n.s.	n.s.	n.s.	n.s.	n.s.	n.s.
**Equatorial (%)**	n.s.	n.s.	n.s.	n.s.	n.s.	n.s.	n.s.	n.s.	n.s.	n.s.	n.s.	n.s.	n.s.
**Acrosomal+equatorial (%)**	n.s.	n.s.	n.s.	n.s.	n.s.	n.s.	n.s.	n.s.	n.s.	n.s.	n.s.	n.s.	n.s.
**Dispersed (%)**	n.s.	n.s.	n.s.	n.s.	n.s.	n.s.	n.s.	n.s.	n.s.	n.s.	n.s.	n.s.	n.s.

Sperm from corresponding human patients undergoing fertility treatment were examined. Statistically significant (*P *≤* *0.05) differences are indicated, along with the corresponding Pearson’s correlation coefficient (*r*; positive values indicate a positive correlation). n.s., non-significant differences; tPNa, time to pronuclear appearance; tPNf, time to pronuclear fading; CC2, time taken between the second and third cell cycle; S2, time taken between the third and fourth cell cycle; t2–t8, time taken to get to number of cells (2–8).

### Relationship between CC2 times in embryos and human sperm PLCζ levels

**Figure 2.**
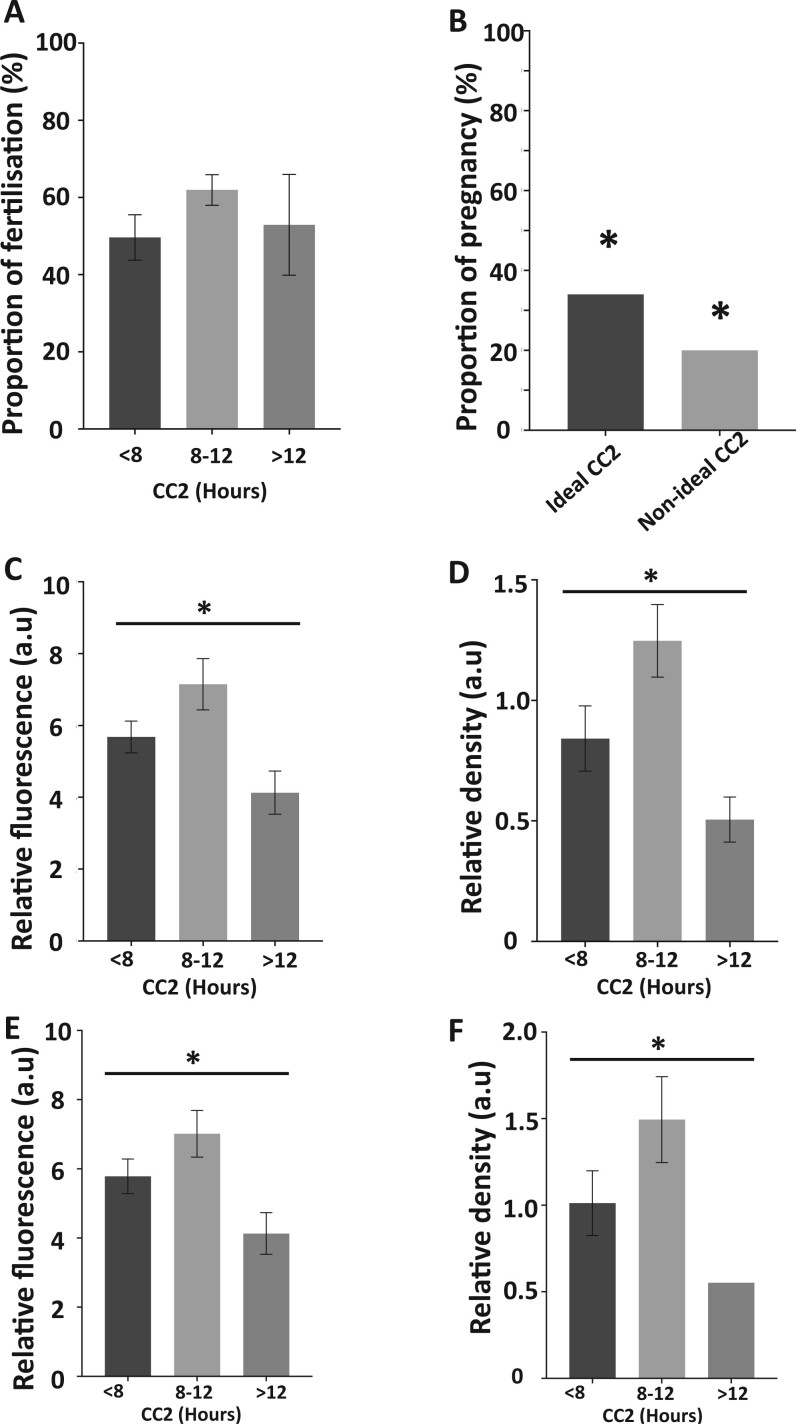
**Histograms indicating relationships of parameters examined in relation to the second cell cycle (CC2).** (**A**) Proportion (%) of successful fertilization in cases exhibiting CC2 times of <8, 8–12, and >12 h. (**B**) Cases with a CC2 of 8–12 h exhibited a significantly higher proportion of successful pregnancy compared to those with non-ideal times (<8 and >12 h). Sperm PLCζ quantification using (**C**) EF pAb relative fluorescence, (**D**) EF pAb relative density, (**E**) I33 mAb relative fluorescence, and (**F**) I33 mAb relative density in relation to CC2 timepoints of <8, 8–12, and >12 h. CC2 of 8–12 h exhibited significantly higher levels of PLCζ compared to non-ideal times. Asterisks (*) indicate a statistically significant (*P *≤* *0.05) difference. Data are indicative of 100 cells examined from 54 patients. a.u: arbitrary units. pAB: polyclonal antibody; mAB: monoclonal antibody; CC2: time between the second and third cell cycles.

Considering the significant relationship between various human sperm PLCζ profiles and CC2, further examination revealed that CC2 time groups did not exhibit a significant difference when compared against proportions of fertilization (Fig. 2A), yet the ideal time for CC2 (8–12 h) exhibited significantly higher proportions of successful pregnancy compared to non-ideal CC2 times, as examined by the χ^2^ test (Fig. 2B). Higher levels of human sperm PLCζ (relative fluorescence and density of 6.5 and 1.25 a.u., respectively) were observed in ideal times of CC2 (8–12 h), compared to ranges outside the ideal timeframe (<8 and >12 h), where levels of human sperm PLCζ were lower. This observation was made using both the EF pAb (Fig. 2C and D) and the I33 mAb (Fig. 2E and F). However, early datapoints of fertilization (namely proportion of fertilized oocytes, tPB2, and t2) did not exhibit such differences in relation to ideal CC2 times (Supplementary Fig. S3).

**Figure 3.**
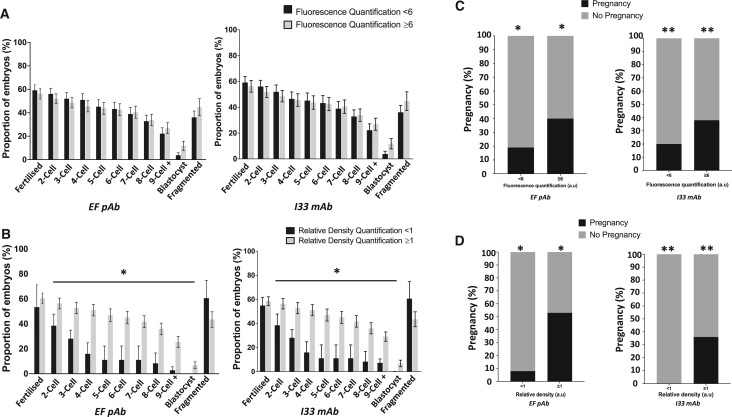
**Histograms indicating the relationship between embryo development in relation to ideal times of CC2 and cut-off values of sperm PLCζ.** Proportions (%) of embryos formed were compared in relation to sperm PLCζ values below and above assigned cut-off of (**A**) relative fluorescence (<6: dark grey or ≥6: light grey) and (**B**) relative density (<1: dark grey or ≥1: light grey). Proportions of pregnancies achieved were also examined in relation to (**C**) relative fluorescence (<6 or ≥6) and (**D**) relative density (<1 or ≥1) cut-off values of PLCζ. Histograms indicate cases of successful pregnancy (dark grey) and no pregnancy (light grey). Asterisks (*) indicate a statistically significant (*P *≤* *0.05) difference. Data are indicative of 100 cells examined from 54 patients. a.u: arbitrary units; pAB: polyclonal antibody; mAB: monoclonal antibody.

From this, we assigned threshold values of PLCζ quantification in human sperm corresponding to the lowest value of PLCζ observed within the ideal CC2 timeframe of 8–12 h (6a.u. for relative fluorescence, and 1a.u. for relative density quantification). While relative fluorescence quantification did not exhibit significant differences throughout all morphokinetic parameters when using both antibodies, significant differences were observed when examining relative density quantification of PLCζ using both antibodies. In cases where sperm PLCζ relative density was ≥1, the proportions of embryos at the 2-cell to blastocyst formation stages were higher compared to that in cases where sperm PLCζ relative density was <1. However, proportions of fertilized and fragmented embryos did not differ (Fig. 3A and B). Interestingly, examination of these thresholds of PLCζ quantification revealed a significantly higher (*P *≤* *0.05) proportion with successful pregnancy in cases of sperm with values larger than the assigned cut-off for both relative fluorescence (19% vs 40%, respectively for EF pAb and 20% vs 42%, respective for I33 mAB; Fig. 3C) and relative density (8% vs 54%, respectively for EF pAB and 0% vs 35% for I33 mAb; Fig. 3D).

**Figure 4.**
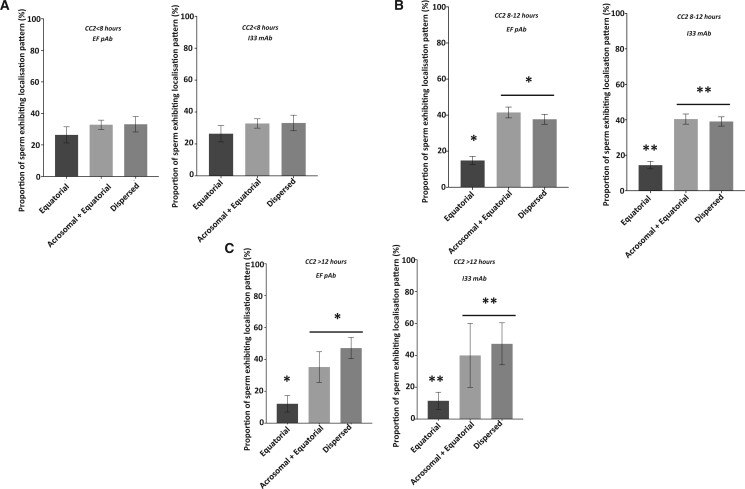
**Histograms showing the proportions of sperm exhibiting different PLCζ localization patterns for cases with different embryo development parameters.** (**A**) Cases with CC2 time <8 h, (**B**) cases with CC2 time 8–12 h and (**C**) cases with CC2 time >12 h. Asterisks (*) indicate a statistically significant (*P *≤* *0.025) difference. Data are indicative of 100 sperm cells examined from 54 cases. a.u: arbitrary units; pAB: polyclonal antibody; mAB: monoclonal antibody; CC2: time between the second and third cell cycles.

**Figure 5.**
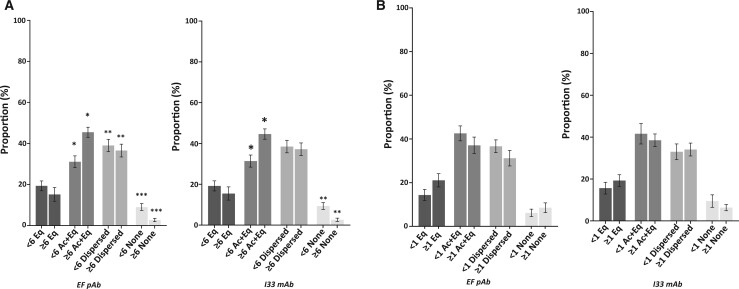
**Histograms indicating the proportions of sperm exhibiting different PLCζ localization patterns.** Cases below or above the assigned threshold for (**A**) PLCζ fluorescence, and (**B**) PLCζ relative density. Asterisks (*, **, ***) indicate a statistically significant (*P *≤* *0.025) difference. Data are indicative of 100 cells (fluorescence quantification) or three repeats (relative density quantification) of sperm examined from 54 patients. a.u: arbitrary units. pAB: polyclonal antibody; mAB: monoclonal antibody; Eq; Equatorial; Ac+Eq: Acrosomal + Equatorial.

Examining PLCζ localization patterns in relation to fertilization indicated that the acrosomal+equatorial and dispersed patterns were highest in cases of successful fertilization as compared to no significant variation between localization patterns in cases of no fertilization (Supplementary Fig. S4). A similar scenario was observed with successful pregnancy cases (Supplementary Fig. S5), in line with previous observations (Kashir *et al.*, 2020, 2023). Acrosomal+equatorial and dispersed localization patterns were most dominant in cases whose embryos exhibited the 8- to 12-h ideal CC2 timeframe. However, there was no significant difference in PLCζ localization pattern proportions in cases with CC2 < 8 h, while acrosomal+equatorial and dispersed patterns were dominant in cases with CC2 > 12 h (Fig. 4). Similarly, examining PLCζ localization in relation to the assigned PLCζ quantification cut-off values also suggested that the acrosomal+equatorial and dispersed localization patterns were most prevalent regardless of levels of PLCζ, but this difference was more profound in values larger or equal to assigned cut-off values for both relative fluorescence and density (Supplementary Fig. S6). Two-way ANOVA suggested that both localization pattern and threshold cut-off (fluorescence and density) were significantly influential in determining the trends observed. However, only fluorescence quantification indicated a significant difference between localization patterns in sperm above the threshold cut-offs compared to those below, with Ac+Eq localization exhibiting higher levels in cases above the threshold compared to below, while dispersed and no patterns decreased in proportion (Fig. 5). No significant difference between localization patterns was observed using the relative density quantification threshold.

### Mutant PLCζ in mouse sperm

**Figure 6.**
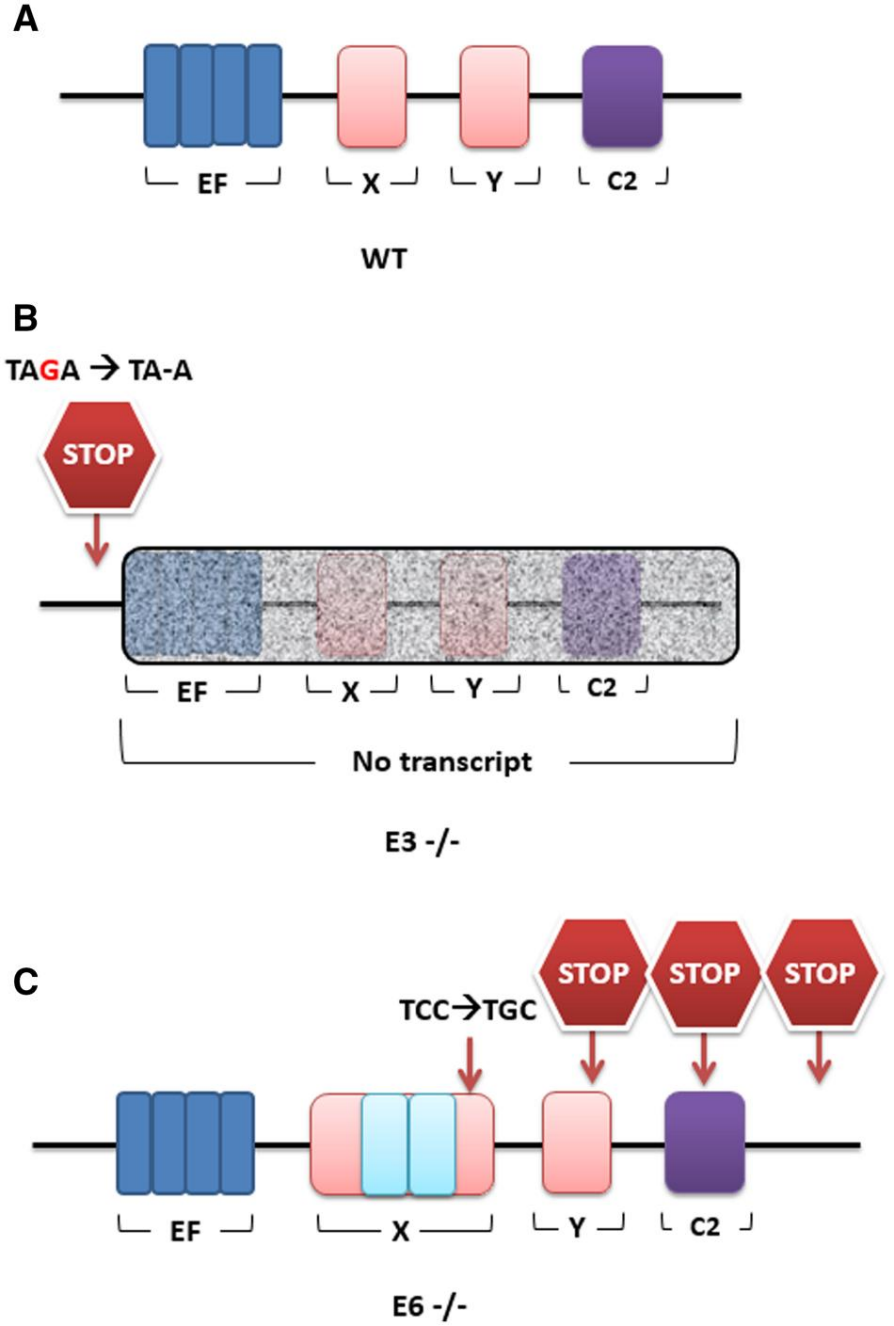
**Schematic representations of the expected distribution of PLCζ domain distribution.** EF hands: blue; X and Y domains: red; and C2 domain: purple, in (**A**) wildtype (WT), (**B**) Exon 3 homozygous (E3−/−), and (**C**) Exon 6 homozygous (E6−/−) strains of mice. E3−/− mice had a single base pair deletion creating a premature stop codon just before the EF hands, resulting in an expected deletion of subsequent domains (shaded box). E6−/− mice had a ×2 insertion of the ssODN donor sequence in the X domain (blue boxes), alongside the envisaged single base pair change in the X domain creating multiple predicted stop codons downstream.

Generation of *Plcζ* mutant mice by the CRISPR/Cas9 system yielded two mutant strains that were used in this study in addition to the WT. One of the *Plcζ* mutant strains had single-nucleotide deletion in E3 (before the EF hands domain) of the *Plcζ* open reading frame causing a frameshift mutation that resulted in generation of premature stop codon (E3; NM_054066.4: c.68delG: p.R48KfsX26), thus causing a predicted premature termination of *Plcζ* translation (Fig. 6). The second *Plcζ* mutant strain resulted from ×2 insertion of the ssODN donor sequence within the E6 target sequence of *Plcζ* causing frameshift mutation (Fig. 6; E6—NM_054066.4: c.674_675ins CACATTCACCAGCAAGCTTCTCTTCAAAAGTTAAATCTCAAGTATGCCTTTGTGGTATGTGTGCGTCTCCGATGCAGACATTTAAAGAATTTACTGCTCCCAAATGCTATACAGCAAGGCCTTACTTTAAATCTGTGGT: p.T235SfsX2) that resulted in the generation of multiple premature stop codons within the X domain coding sequence of *Plcζ.*

Direct sequencing of the exon 3 target sequence from E3 mutant mice confirmed presence of the desired deletion (Supplementary Fig. S7), while direct sequencing of the exon 6 target sequence from E6 mutant mice confirmed presence of the ssODN donor sequence insertion, resulting in a longer PCR fragment amplification of the target sequence in E6 mutant mice compared to the target sequence in WT mice This was also confirmed by agarose gel electrophoresis of genotyping amplicons (Supplementary Fig. S8). Histological evaluation of testicular and epididymal sections did not indicate any significant difference in overall tissue integrity and spermatogenesis between WT and mutant mice (Supplementary Fig. S9), nor did sperm examinations reveal any significant changes in morphology or observable motility/concentration (data not shown).

**Figure 7.**
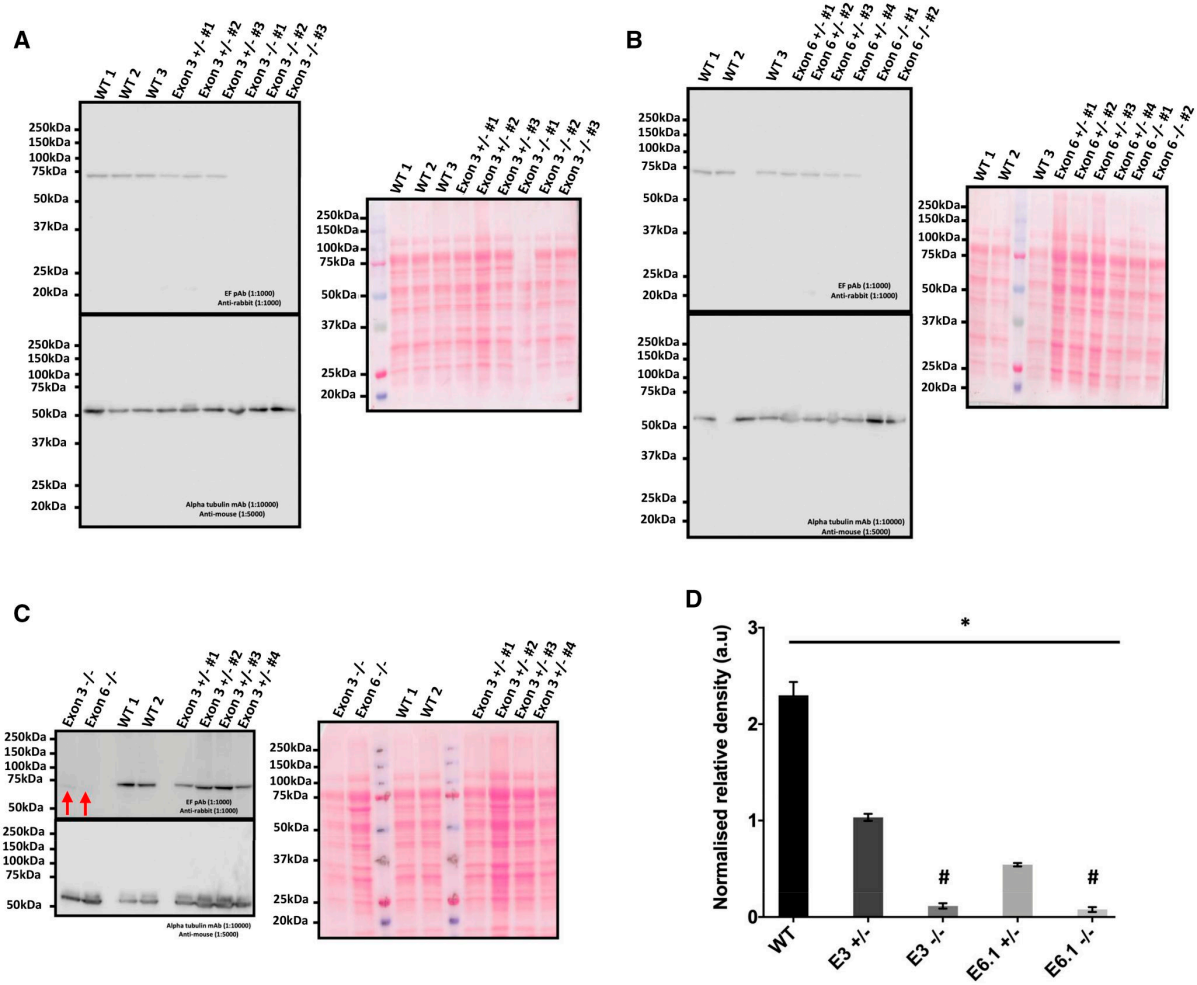
**Immunoblotting analysis and corresponding ponceau-stained images of PLCζ** (**A**) sperm from wildtype (WT), E3 heterozygous (+/−), and homozygous (−/−) mice; (**B**) sperm from WT, E6 heterozygous (+/−), and homozygous (−/−) mice; and (**C**) high exposure of sperm from WT, E3 heterozygous (+/−), and homozygous (−/−) mice indicating a lower band and significantly reduced density present in a minority of E3 (−/−) sperm (red arrows). (**D**) Histogram representing the average quantified relative density of bands observed in sperm from WT, E3 (+/−) and E3 (−/−), and E6 (+/−) and E6 (−/−) strains. Sperm from both (+/−) strains exhibited a significantly reduced level of PLCζ in sperm, with sperm from both (−/−) strains also exhibiting a near negligible (but still present) level of PLCζ, which was insignificant between E3 and E6 strains. Asterisk (*) indicates a statistically significant (*P *≤* *0.05) difference, while hash (#) indicates a non-significant difference (*P *>* *0.05). Data is representative of at least three biological and three technical (3×3) repeats for each strain examined. pAB: polyclonal antibody; mAB: monoclonal antibody.

Immunoblotting analysis detected a single band at 74 kDa corresponding to PLCζ in sperm lysates from WT mice as previously described (Kashir *et al.*, 2021). A single band was also observed in sperm lysates from heterozygous (+/−) E3 and E6 mutant mice (Fig. 7A and B), but at a diminished intensity compared to WT. Sperm lysates from the homozygous (−/−) E6 mutant strain did not exhibit observable bands compared to WT, similar to most sperm lysates from −/− E3 mutant mice. However, in some cases, overexposure of immunoblotting development (saturated WT and +/− bands) indicated a comparatively diminished single band in E3^−/−^ mutant sperm (Fig. 7C). This was confirmed by relative densitometry which indicated a statistically significant reduction in +/− and −/− sperm. The difference between levels of PLCζ in sperm from E3 and E6 −/− was non-significant (Fig. 7D).

**Figure 8.**
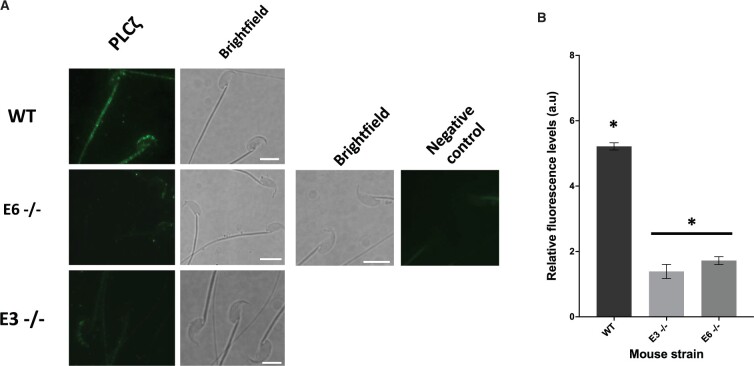
**Representative PLCζ immunofluorescence microscopy in mouse sperm.** (**A**) Observed PLCζ localization patterns (green fluorescence; left column) alongside corresponding brightfield images (right column) in sperm from wildtype (WT; top panel), E6 homozygous (E6−/−; middle panel), and E3 homozygous (E3−/−; bottom panel) strains of mice. Sperm were also treated in the absence of primary antibody to give negative control images (far right panel). Representative images obtained were captured at 100×, while white scale bars represent 10 μm. Images are representative of 100 cells examined for each group. (**B**) Histogram indicating quantification of PLCζ fluorescence in sperm from WT, E3 (−/−) and E6 (−/−) strains of mice. Asterisk (*) indicates a statistically significant (*P *≤* *0.05) difference. Data are indicative of 100 sperm cells examined from each strain. a.u: arbitrary units.

Immunofluorescent analysis indicated previously reported (Kashir *et al.*, 2021) fluorescence patterns in WT sperm, with fluorescence detected in acrosomal and post-acrosomal regions of the sperm head, alongside fluorescence observed in the tail (Fig. 8A). Sperm from both −/− E3 and E6 mutant strains exhibited a significantly diminished and dispersed, punctate pattern, predominantly at the acrosomal region of the sperm head. Interestingly, no fluorescence was observed at the sperm tail (Fig. 8A). Relative fluorescence quantification indicated a significantly reduced level of PLCζ fluorescence in −/− sperm from both E3 and E6 mutant strains compared to the WT, with no significant difference observed between −/− E3 and E6 sperm (Fig. 8B).

### Mutant mouse breeding and embryogenesis

**Figure 9.**
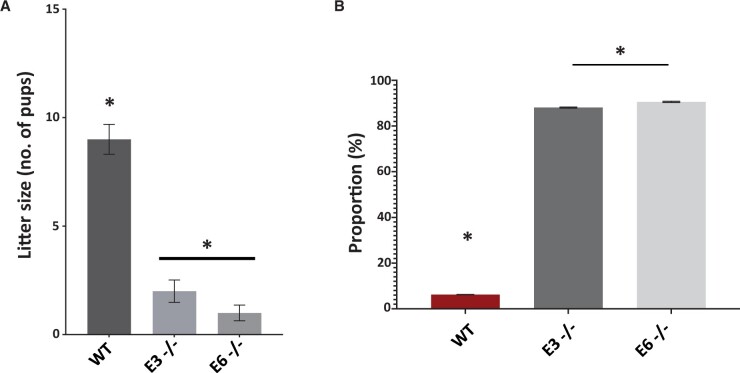
**Histograms representing reproductive outcomes in male mice.** Analysis included wildtype (WT), E3 homozygous (E3−/−), and E6 homozygous (E6−/−) strains of mice. (**A**) Average litter size obtained following breeding of wildtype (WT) females with males of WT, E3(−/−), and E6(−/−) strains. (**B**) The proportion of embryos exhibiting polyspermy following IVF experiments using oocytes from WT females and sperm from WT, E3(−/−) and E6(−/−) males. Asterisk (*) indicates a statistically significant (*P *≤* *0.05) difference, and data are representative of three biological and three technical (3×3) repeats.

Breeding experiments indicated that WT mice yielded an average litter size of 8–10 pups, while E3^−/−^ and E6^−/−^ male mice breeding with WT females yielded a significantly reduced litter size of one to three pups (Fig. 9A). IVF experiments using sperm from WT, E3^−/−^ and E6^−/−^ mice and oocytes from WT females, indicated that while WT sperm yielded a fertilization rate of ∼80%, sperm from E3^−/−^ and E6^−/−^ mice yielded significantly reduced fertilization rates of ∼43% and ∼30% respectively (Supplementary Fig. S10A). This trend was observed throughout all embryogenic developmental timepoints examined, with embryos produced using WT sperm consistently exhibiting higher rates of 2-cell to blastocyst stages of embryonic development compared to those produced with E3^−/−^ and E6^−/−^ sperm. Interestingly, E6^−/−^ mice yielded a higher rate of embryogenesis at all developmental milestones compared to the E3^−/−^ strain. Examination of time-lapse imaging of mouse embryos indicated that those produced by WT and E6^−/−^ mice exhibited similar time periods for embryogenesis while those of the E3^−/−^ strain took significantly longer for cell divisions to occur (Supplementary Fig. S10B). However, the analysis also revealed a significantly high proportion of polyspermy in embryos generated by E3^−/−^ and E6^−/−^ compared to WT males (82%, 84%, and 10%, respectively) (Fig. 9B).

**Figure 10.**
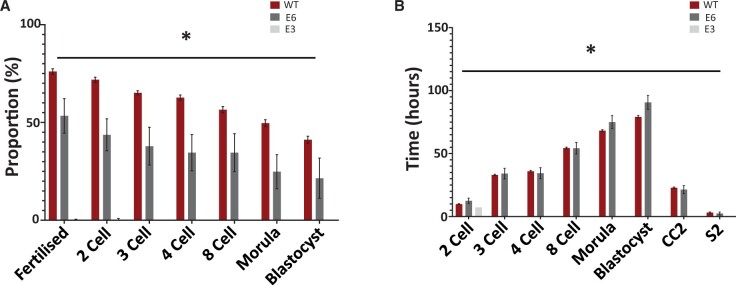
**Histograms representing embryogenic efficacy of embryos generated following IVF)** Experiments used oocytes from wildtype (WT) females and sperm of WT (red), E3 homozygous (E3−/−; dark grey), and E6 homozygous (E6−/−; light grey) strains of mice (excluding cases of polyspermy), indicating (**A**) proportions of embryos generated at specific developmental milestones, and (**B**) time taken (h) to reach specific developmental morphokinetic milestones. Asterisk (*) indicates a statistically significant (*P *≤* *0.05) difference, and data are representative of three biological and three technical (3×3) repeats. CC2: time taken for second cell cycle (2-cell to 3-cell); S2: time taken for third cell cycle (3-cell to 4-cell).

Removing polyspermic embryos from our analyses indicated that the E3^−/−^ strain exhibited a severely reduced level of fertilization (∼1%) compared to WT. While E6^−/−^ mice exhibited higher levels of fertilization compared to the E3^−/−^ strain (53%), it was still significantly reduced compared to WT (77%). Similar trends were observable for proportions of embryos reaching various milestones, with E6^−/−^ mice generating a significantly reduced proportion of embryos at each stage compared to WT, while E3^−/−^ males produced embryos that did not develop past the 2-cell stage (Fig. 10A). Time-lapse analysis revealed no significant difference between embryos produced by WT and E6^−/−^ sperm in terms of time taken for early cell divisions (2-cell, CC2, S2, 4-cell, and 8-cell times). The embryos from the E3^−/−^ strain of mice were quicker to reach the 2-cell stage, but no other cell divisions were observable, while the embryos produced by the E6^−/−^ males took a significantly longer time to reach the morula and blastocyst stages compared to WT embryos (taking 3 h longer and 5 h longer, respectively) (Fig. 10B).

## Discussion

While embryogenesis has been long considered to be exclusively a maternal effect, studies have suggested that multiple paternal factors can influence embryogenesis. In their recent review, Vallet-Buisan *et al.* (2023) discussed various potential male factors that could exert such a role including the paternal centriole, various RNA transcripts and proteins, DNA integrity and epigenetic changes that could impact fertilization as well as early stages of embryo development. Indeed, poor embryogenesis has been previously attributed to poor sperm parameters (Loutradi *et al.*, 2006; Knez *et al.*, 2011), while we have recently demonstrated strong links between PLCζ profiles and sperm parameters in human patients, as well as showing that increasing age is associated with poor PLCζ profiles in mouse sperm (Kashir *et al.*, 2020, 2021, 2023). Given the significant beneficial potential for PLCζ as a therapeutic intervention in cases of male infertility where PLCζ is significantly reduced or absent, herein we examined for the first time whether specific profiles of PLCζ could be linked to clinical indicators of embryogenesis in both humans and mouse.

### Oocyte activation, calcium oscillations and embryo morphokinetics

We observed that the times taken for CC2 corresponded to successful pregnancy in line with the published literature, while no specific relationship was found between CC2 times and fertilization success. However, we could not find a correspondence between CC2 and other morphokinetic parameters of fertilization including the time taken for second polar body extrusion or the duration of the 2-cell stage. Traditionally, these have been thought to be linked to the efficacy of oocyte activation, and by association PLCζ, as the frequency and amplitude of Ca^2+^ oscillations can alter the rate of progression of both these parameters (Ducibella *et al.*, 2002, 2006). However, it was apparent that there was no specific link between PLCζ and these parameters of fertilization in humans, perhaps indicating that while there is variance in levels of human sperm PLCζ, this variance does not sufficiently alter rates of these morphokinetic parameters. We could not examine these early parameters in our mouse IVF experiments due to the protocols employed and requirement to synchronize at a specific timepoint to compare between strains. It is thus necessary that similar experiments be performed to ascertain the nature of such links.

Consensus indicates that CC2 and S2 (within specific ranges) are particularly informative regarding embryo quality and implantation (Wong *et al.*, 2010; Meseguer *et al.*, 2011; Milewski and Ajduk, 2017), with embryos falling outside of recommended ranges perhaps representing suboptimal cytoplasm/nuclear machinery within early blastomeres, which are passed on to descendant blastomeres, compromising embryo viability. Indeed, Yang *et al.* (2015) suggest that earlier irregular cleavages exert greater their impact upon embryogenic efficacy. Importantly, ideal morphokinetic parameters strongly favour embryos which arise from an ideal cell cycle progression in all blastomeres (Milewski and Ajduk, 2017). Perhaps most relevant to this is that a specific range of PLCζ delivered to the oocyte can effectively elicit the required Ca^2+^ oscillations and result in oocyte activation progression in mice and humans (Cox *et al.*, 2002; Saunders *et al.*, 2002; Yamaguchi *et al.*, 2017), which are estimated to be approximately double (20–40 fg) in mouse (Kashir *et al.*, 2022; Abdulsamad *et al.*, 2023), indicating a wide range of variability of sperm PLCζ that could activate with varying efficacies of cell cycle progression.

### Minimum levels of PLCζ are required for effective embryogenesis and pregnancy

Our results indicate for the first time a link between levels or localization patterns of PLCζ delivered by the sperm to the oocyte and indicators of embryogenic health as well as successful pregnancy when examined in the context of timelapse-imaging (TI) of embryos. TI systems, wherein images of embryogenesis are recorded at regular intervals in an incubator, are widely employed by fertility clinics globally with significant data now reported following its use. While specific fertilization parameters were associated with embryo quality on Day 3 (Coticchio *et al.*, 2018), longer times of zygotic pronuclear fading were associated with higher live births (Azzarello *et al.*, 2012). However, we did not observe any significant correlation between early fertilization and embryogenic competency.

While evidence does not suggest that embryo TI can improve pregnancy rates compared with standard incubation/assessment, embryo implantation is associated with specific cell division timing parameters (Apter *et al.*, 2020). The timing of the five-cell cleavage and durations of the two-cell and three-cell stages (t5, s2, cc2) exhibited the most predictive value for embryo viability and implantation (Wong *et al.*, 2010; Meseguer *et al.*, 2011; Apter *et al.*, 2020). While we could not ascertain significant correlations between PLCζ parameters and t5, we did observe a significant positive relationship between PLCζ levels and CC2 as quantified by two methods, and a significant relationship with relative fluorescence quantification of PLCζ and S2.

It is worth noting that our Pearson correlation models exhibited relatively lower r^2^ values, but with significant *P*-values. In ideal scenarios, one would expect r^2^ values to be approaching (or as close as possible to) ±1, with higher values for r^2^ exhibiting stronger correlation models. However, this does not necessarily mean that lower r^2^ values correspond to weaker or less significant models, but rather that data exhibiting a high degree of variability has been used to construct this model, as is usually the case with data obtained from clinical fertility treatment. Of course, even in cases that r^2^ values are approaching ±1, this does not necessarily mean that one variable causes the other, but rather the variables are associated (Armitage *et al.*, 2002; Bland, 2015). Given that the relationships we focus on are statistically significant (namely with CC2, S2, and KIDScores), we can say that there is a relationship which is worth investigating further, enabling us to examine these relationships in more detail, as we did subsequently.

CC2 time ranges specifically have been widely studied, with a consensus of studies indicating that ideal timings of CC2 range between 8 and 12 h in humans (Desai *et al.*, 2014; Kovacs, 2014; Tejera *et al.*, 2016; Barrie *et al.*, 2017; Meng Ju, 2017; Apter *et al.*, 2020; Sayed *et al.*, 2020). Interestingly, our data indicated that in sperm from cases where embryos exhibited CC2 within 8–12 h possessed higher PLCζ levels compared to times out of this range. We also observed that a CC2 of 8–12 h range also exhibited a significantly higher number of pregnancies compared to the other timepoints.

Given the prognostic value of CC2, we assigned threshold values of PLCζ levels (6 a.u. for relative fluorescence and 1 a.u. for relative density). We found that the relative fluorescence threshold did not yield any significant differences in proportions of embryos reaching developmental milestones. However, cases with sperm PLCζ relative density ≥ 1 corresponded to a significantly higher proportion of embryos reaching the 2-cell to blastocyst stages. Astoundingly, we observed that sperm PLCζ ≥threshold levels exhibited a significantly higher proportions of successful pregnancy (∼2-fold and ∼5.5-fold levels higher using fluorescence or density quantification, respectively). To this degree, it seems that minimal levels of PLCζ are required within sperm to ensure embryogenic competency leading to pregnancy.

Interestingly, we also observed that PLCζ levels may also be related to measure of embryogenic quality as indicated by the KIDScore. As an additional evaluation algorithmic tool specific to the ‘Embryoscope’ TI system, the KIDScore provides an additional predictive evaluation of implantation, ranking embryos (one to five on earlier models) based upon morphological criteria, with higher KIDScores generally corresponding to embryos with highest implantation potential, pregnancy, and live birth (Reignier *et al.*, 2019; Tartia *et al.*, 2022). We found that levels of PLCζ were positively correlated with the highest KIDScore group examined, although we could not ascertain the specific nature of this correlation, and this may differ on more modern/updated version of algorithms.

A similar scenario was observed in our mutant mouse experiments whereby our breeding experiments using *Plcζ* mutant males with WT females showed a severe reduction in litter size. This correlated with the reduction of PLCζ in mouse sperm, indicating a reducing efficacy of live birth with reducing levels and abnormal localization of sperm PLCζ. This abnormal profile of sperm PLCζ also corresponded to a high proportion of polyspermy in IVF experiments using homozygous mutant males compared to WT males. Interestingly, this also corresponded to a reduction in the proportion of embryos generated throughout developmental milestones in both homozygous strains, with E3^−/−^ embryos not proceeding past the 2-cell stage when accounting for polyspermic embryos. Interestingly, embryos generated using E6^−/−^ sperm that were able to develop did so in similar morphokinetic parameters compared to WT, but took longer to develop to morula and blastocyst compared to WT.

Collectively, these results indicate a similar scenario to that in humans, whereby a reduction of PLCζ results in successful fertilization, but reduction in efficacy of embryogenesis and severe reductions in pregnancy. Our work is in concordance with previous studies (Hachem *et al.*, 2017; Nozawa *et al.*, 2018; Hirose *et al.*, 2023; Wang *et al.*, 2023) that indicated severely high polyspermy, reduced proportions of fertilization and proportions of embryogenic milestones reached by embryos generated with PLCζ mutant sperm. However, while our breeding experiments also yielded similar data, the embryogenic efficacy of E3^−/−^ mice was absent beyond the 2-cell stage, with our data also indicating for the first time differences in morphokinetic parameters in embryos generated by PLCζ mutant sperm compared to WT.

Generally, we did not observe specific correlations between localization patterns of PLCζ and embryogenic parameters examined. Perhaps this reflects our previous assertions that while the acrosomal+equatorial pattern is more physiologically relevant to sperm quality (Kashir *et al.*, 2021, 2023), levels of PLCζ are more relevant to embryogenic efficacy rather than localization. Indeed, we generally observed in both human and mouse that only a minority of sperm exhibited completely absent levels of PLCζ which was only the case in human sperm (‘None’ localization pattern), with mutant mouse sperm able to present with some (abnormal) PLCζ localization. Indeed, our negative controls and specificity of immunoblotting suggest that these patterns are likely not a background artefact.

### PLCζ in mutant mouse sperm is severely reduced, but not absent

Most intriguingly, our data shows that PLCζ was not entirely absent in mutant mouse sperm. While most immunoblotting experiments indicated a relative absence of a band corresponding to mouse PLCζ, longer exposures indicated presence of a severely diminished PLCζ band in mutant sperm compared to WT. Furthermore, our immunofluorescence experiments also indicated a punctate pattern and severely reduced levels of PLCζ in mutant sperm. While most of our breeding and embryogenic mutant mouse data is similar to previous studies (Hachem *et al.*, 2017; Nozawa *et al.*, 2018; Hirose *et al.*, 2023; Wang *et al.*, 2023), a major difference apart from the embryo morphokinetic analysis, is the extent of sperm PLCζ analysis. Apart from Hirose *et al.* (2023) who did not confirm PLCζ in mutant sperm, all previous studies generating PLCζ mutant mice typically attempted to ascertain PLCζ status in mutant mice using genotyping, reverse-transcription polymerase chain reaction (RTPCR) and immunoblotting. However, while genotyping and RTPCR would ascertain presence of inserted mutations, this would not fully ascertain the complete absence of PLCζ in sperm without relative quantification. While this could be said to have been attempted by immunoblotting, there is significant concern regarding antibody specificity in these studies. Indeed, full immunoblotting profiles of WT and mutant sperm lysates by these studies indicate either a significant number of other bands also identified by the antibodies used, or bands at a slightly lower molecular weight than expected in mouse sperm.

To this degree, none of the previous studies can fully assert with confidence that PLCζ is completely absent from mutant sperm. This is relevant as given the supreme sensitivity of PLCζ in eliciting Ca^2+^ release, even minor levels of PLCζ can lead to reduced patterns of Ca^2+^ oscillations, as was observed previously (Hachem *et al.*, 2017; Nozawa *et al.*, 2018). Perhaps due to such concerns regarding antibody specificity, none of the previous transgenic studies attempted to ascertain PLCζ localization in mutant sperm, in stark contrast to the current study where our immunofluorescence data is in concordance with immunoblotting data regarding severely reduced, but not completely absent, levels of PLCζ. Indeed, the antibody used in the current study has been extensively used with consistent results throughout studies, and extensively validated in both mouse and human sperm (Yu *et al.*, 2012; Nomikos *et al.*, 2013, 2015b, 2017; Kashir *et al.*, 2016, 2020, 2021, 2023). It would be interesting to ascertain the specific reasons underlying the failure to completely delete levels of PLCζ within these mice. However, the significantly reduced profile of PLCζ within sperm from these two strains serves the purposes of the current study whereby we wanted to examine the effect of PLCζ reduction in sperm leading to successful fertilization, but ineffective embryogenesis.

This is perhaps directly relevant to recent assertions regarding the potential impact of severe subfertility, but not infertility, upon the mechanisms of oocyte activation and the pivotal role of sperm PLCζ in this process. Suggestions have been made regarding an alternative, additional or indirect sperm factor in mice, which could perhaps act either in conjunction with PLCζ or may act as a redundancy mechanism (Jones, 2018; Swann, 2020; Jones *et al.*, 2022), which could be perhaps another sperm PLC given the required PIP_2_/IP_3_ dependent mechanisms involved. Indeed, our data support such assertions, as although PLCζ was severely diminished, a comparative rate of fertilization and early embryo morphokinetics were observed between WT and E6^−/−^ mice. However, this was completely different in E3^−/−^ mice, which predominantly exhibited severely reduced fertilization rates, and those that did fertilize did not proceed beyond the 2-cell stage in IVF experiments. Furthermore, breeding experiments indicated that pups were produced from mutant sperm. Given the above, the assertion of additional mechanisms involved in maintaining oocyte activation does carry credence, but perhaps remains dependent upon PLCζ, at least in mice.

A good indication of this in our results are the proportion of embryos produced and corresponding morphokinetics in embryos generated from PLCζ mutant sperm when including polyspermic embryos. Considering that our results indicated a severely reduced profile of PLCζ in mutant sperm, polyspermy would have meant larger levels of PLCζ being delivered to the oocyte, thus allowing cell cycle progression and embryogenesis, albeit abnormal. This resulted in a larger number of embryos reaching developmental milestones, but still taking longer times in morphokinetic parameters in the E3^−/−^ strain of mouse, suggesting that additional levels of PLCζ would be able to allow progression of embryogenesis.

Given our results, it is worth cautioning that while we took a number of steps to control these, several parameters may influence embryonic developmental capacity in humans. Generally, we included males who exhibited a suitable level of sperm that could be used for both fertility treatment as well as our experiments (i.e. a minimum sperm concentration of 5×10^6^ sperm/ml). While several male factor conditions were included in our recruitment (Supplementary Table S4), we could not find any further correlations with the embryogenic parameters examined and sperm data extracted. While a whole range of maternal factors are also known to affect embryogenesis (maternal mutations/oocyte mRNA, etc.), it was not possible to assess these within the ethical clearances granted to the study, as well as the fact that we needed to ascertain the embryogenic capacity of such oocytes/embryos, excluding our ability to perform molecular analyses on these samples. We also made efforts to not include cases where maternal factors such as specific mutations or defects were associated with cases (which would already have been limited by our five oocytes per cycle criteria). To this degree, it was perhaps more important to perform similar experiments on a more controlled mouse model as we have done. However, it is also worth noting that the major cytoplasmic determinant affecting at least the early cell cleavage is the oocyte cytoskeleton, specifically the actomyosin cytoskeleton that is itself susceptible to influence by sperm-induced Ca^2+^ oscillations (Gray *et al.*, 2004; Ajduk *et al.*, 2011; D’Avino *et al.*, 2015; Milewski *et al.*, 2018).

The outcomes of both human and transgenic experiments support our assertion that profiles of sperm PLCζ may underlie quality of embryogenesis beyond fertilization. Indeed, our human data suggested that levels of sperm PLCζ below a certain threshold corresponded to poor embryogenesis and low pregnancy despite resulting in successful fertilization. We also confirmed that abnormal transgenic mouse sperm PLCζ led to a similar scenario to the human data where abnormal/reduced sperm PLCζ resulted in slower rates of embryogenic progression, and overall lower proportions of embryonic milestones and reduced litter sizes. In this aspect, the time-lapse imaging of the transgenic-induced embryos (of both strains) are also a novel aspect of our study. Indeed, this is further illustrated by our findings that sperm from our E3−/− strain that successfully fertilized (excluding cases of polyspermy) did not yield embryos beyond the 2-cell stage, supporting our assertions that reduced levels of PLCζ could result in successful fertilization, but a specific amount is perhaps required to cause meaningful embryogenesis. However, more in-depth investigations are now required by subsequent studies to examine this phenomenon in more detail such as examination of multiple embryogenic cycles of the same couple, which was beyond the scope of our current ethical clearance.

Despite some supportive connectivity between results obtained, the mouse and human data we present are, in essence, independent analyses. Indeed, the variability in PLCζ protein levels we observed in mouse sperm could be attributed to mutations inserted following CRISPR/Cas9 methodology. However, the variability in human sperm PLCζ protein levels remains unaccounted for. It would be invaluable for future studies to perform screening of the *Plcζ* gene in humans concurrent to analyses performed in the current study to ascertain whether a causative genetic factor was also involved. Indeed, it is already well established in the literature that identified clinically relevant *Plcζ* mutations also correspond to reduced/absent levels of PLCζ protein in sperm. Finally, while it is well accepted that immune-based methods of PLCζ analysis in sperm are currently the most effective and physiologically relevant, perhaps it would be worthwhile to utilize additional methods of PLCζ quantification other than those utilized by our current study. We used both immunofluorescence and immunoblotting to quantify sperm levels of PLCζ, with both quantification methods exhibiting similar trends and outcomes in both mouse and human. However, it may also be worth considering *Plcζ* RNA quantification of sperm in the context of this study. While the physiological relevance of such analyses may be questionable due to the unclear role of sperm *Plcζ* RNA, this may provide some indicator of sperm/spermatogenesis quality in the context of sperm PLCζ protein levels and quality of embryogenesis.

## Conclusions

To our knowledge, this represents the first time that PLCζ levels in sperm have been correlated to prognostic measures of embryogenic efficacy and pregnancy rates in humans, although this should be further investigated in a larger multi-centre cohort. Generally, our data indicates that PLCζ mutant sperm exhibited severely reduced, but not absent levels of PLCζ, which resulted in a reduction of litter sizes in mutant mice, as well as a reduction of embryos reaching specific developmental milestones, although this differed between strains of mutant mice examined. Specifically, we showed, in both mouse and humans, that minimal levels of PLCζ within specific a certain range were required for optimal embryogenesis and pregnancy. While current cases of ICSI failure are treatable by assisted oocyte activation protocols, these have traditionally been reserved for cases of complete absence or severe reduction of sperm PLCζ. Our data suggests for the first time that the clinical utilization of PLCζ may stand to benefit, not just a specific population of male infertility cases where oocyte activation is completely deficient (wherein PLCζ is completely defective/abrogated), but also perhaps the larger population of couples seeking fertility treatment.

## Supplementary Material

deae078_Supplementary_Figure_S1

deae078_Supplementary_Figure_S2

deae078_Supplementary_Figure_S3

deae078_Supplementary_Figure_S4

deae078_Supplementary_Figure_S5

deae078_Supplementary_Figure_S6

deae078_Supplementary_Figure_S7

deae078_Supplementary_Figure_S8

deae078_Supplementary_Figure_S9

deae078_Supplementary_Figure_S10

deae078_Supplementary_Table_S1

deae078_Supplementary_Table_S2

deae078_Supplementary_Table_S3

deae078_Supplementary_Table_S4

deae078_Supplementary_Table_S5

## Data Availability

The data underlying this article cannot be shared publicly due to the conditions of the ethical approval obtained and to protect the privacy of individuals who participated in the study. The data will be shared on reasonable request to the corresponding author.
