# Supplementary material for: The mammalian sperm factor phospholipase C zeta is critical for early embryo division and pregnancy in humans and mice
Source: Hum Reprod. 2024 Apr 26;39(6):1256–74. doi: 10.1093/humrep/deae078 (PMC11145019; doi:10.1093/humrep/deae078)
Supplement: deae078_Supplementary_Figure_S1 [file deae078_supplementary_figure_s1.pdf]

Human Reproduction, 2024, 1–1
https://doi.org/10.1093/humrep/deae078
Supplementary Data

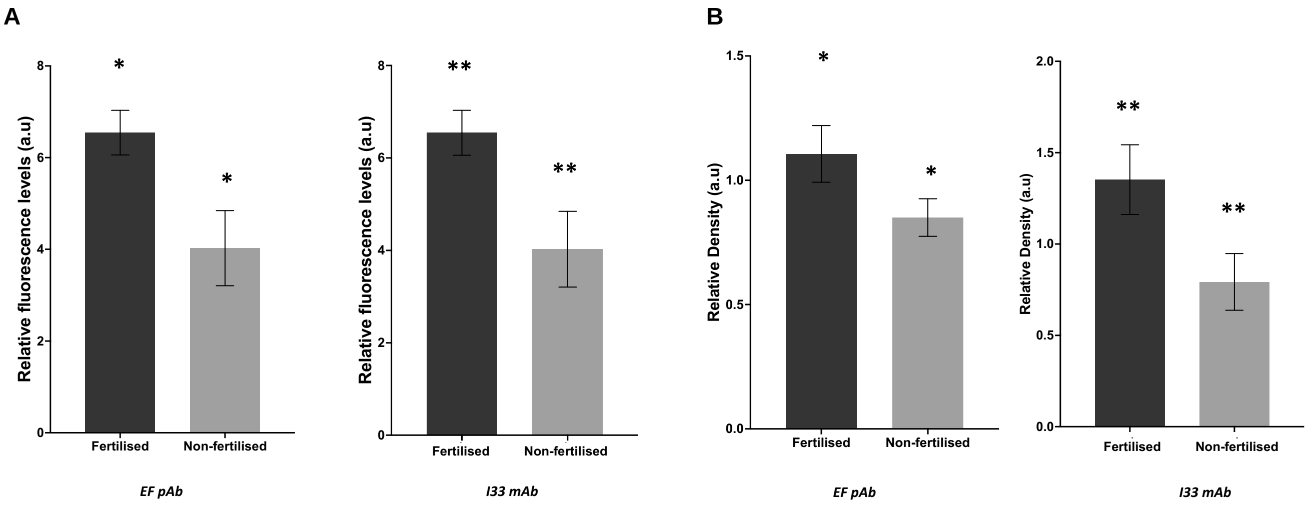

Supplementary Figure S1. Histograms representing the proportion of fertilized and not fertilized oocytes from corresponding human males in relation to levels of PLCζ in sperm as quantified by (A) relative fluorescence and (B) relative densitometry. Asterisks (*) indicate a statistically significant ($P \leq 0.05$) difference. Data are indicative of 100 cells (fluorescence quantification) or three repeats (relative density quantification) of sperm examined from 54 patients. a.u, arbitrary units.
