# Supplementary material for: The mammalian sperm factor phospholipase C zeta is critical for early embryo division and pregnancy in humans and mice
Source: Hum Reprod. 2024 Apr 26;39(6):1256–74. doi: 10.1093/humrep/deae078 (PMC11145019; doi:10.1093/humrep/deae078)
Supplement: deae078_Supplementary_Figure_S3 [file deae078_supplementary_figure_s3.pdf]

OXFORD

human reproduction

Human Reproduction, 2024, 1–1

https://doi.org/10.1093/humrep/deae078

Supplementary Data

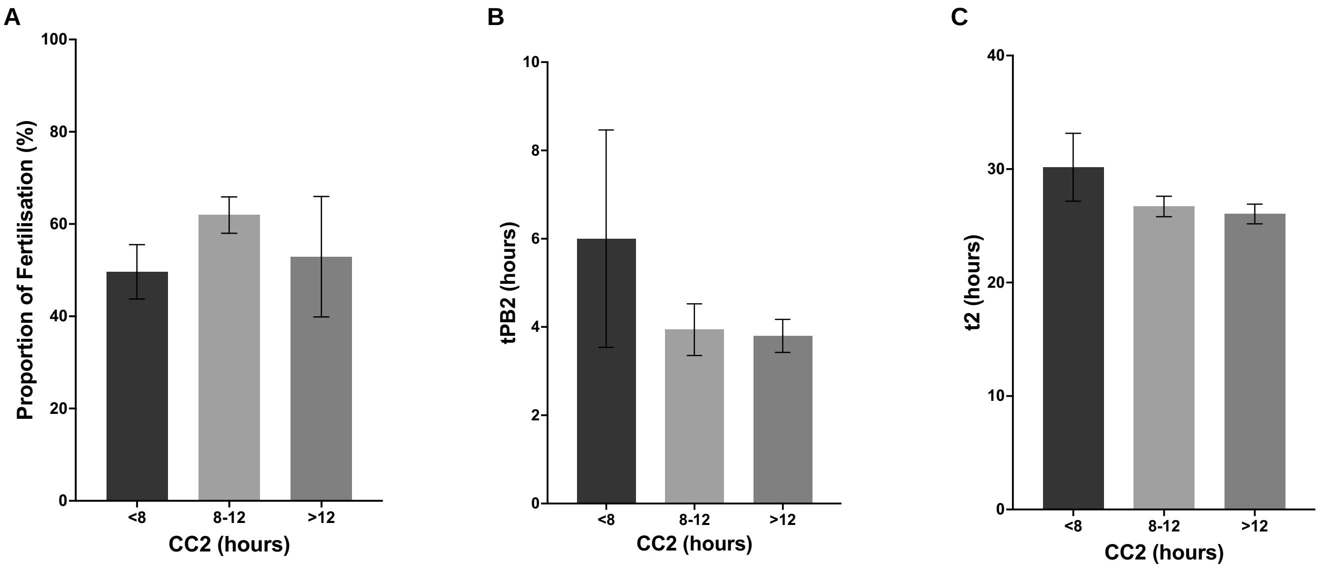

Supplementary Figure S3. Histograms representing the relationship between time taken for CC2 (<8, 8–12, and >12 h) with (A) proportions of fertilization, (B) time taken for second polar body extrusion, and (C) time taken to reach the 2-cell stage. No significant differences were observed. Data are indicative of 54 cases examined for this study.
