# Supplementary material for: The mammalian sperm factor phospholipase C zeta is critical for early embryo division and pregnancy in humans and mice
Source: Hum Reprod. 2024 Apr 26;39(6):1256–74. doi: 10.1093/humrep/deae078 (PMC11145019; doi:10.1093/humrep/deae078)
Supplement: deae078_Supplementary_Figure_S4 [file deae078_supplementary_figure_s4.pdf]

OXFORD
human reproduction
Human Reproduction, 2024, 1–1
https://doi.org/10.1093/humrep/deae078
Supplementary Data

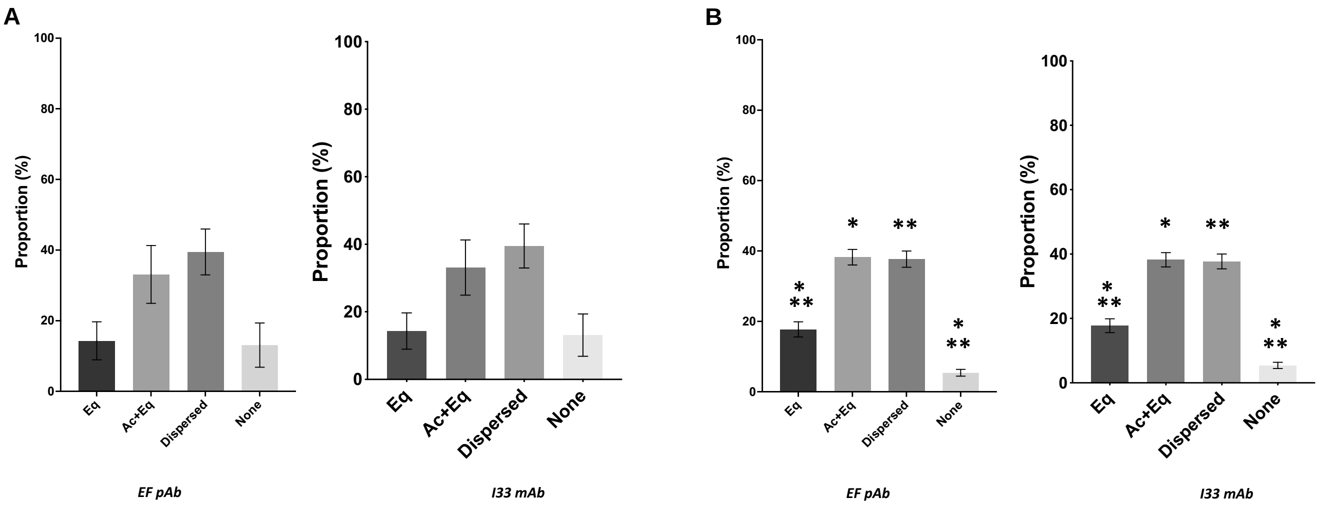

Supplementary Figure S4. Histograms representing the proportion of predominant localization patterns of PLCζ (Eq: equatorial; Ac+Eq: acrosoma+equatorial; dispersed; and none) exhibited by sperm from males involved in cases exhibiting (A) no fertilization and (B) successful fertilization. Asterisks (*) indicate a statistically significant ($P \leq 0.05$) difference. Data are indicative of at 100 sperm cells examined from each of 54 cases examined for this study.
