# Supplementary material for: The mammalian sperm factor phospholipase C zeta is critical for early embryo division and pregnancy in humans and mice
Source: Hum Reprod. 2024 Apr 26;39(6):1256–74. doi: 10.1093/humrep/deae078 (PMC11145019; doi:10.1093/humrep/deae078)
Supplement: deae078_Supplementary_Figure_S6 [file deae078_supplementary_figure_s6.pdf]

OXFORD
human reproduction
Human Reproduction, 2024, 1–1
https://doi.org/10.1093/humrep/deae078
Supplementary Data

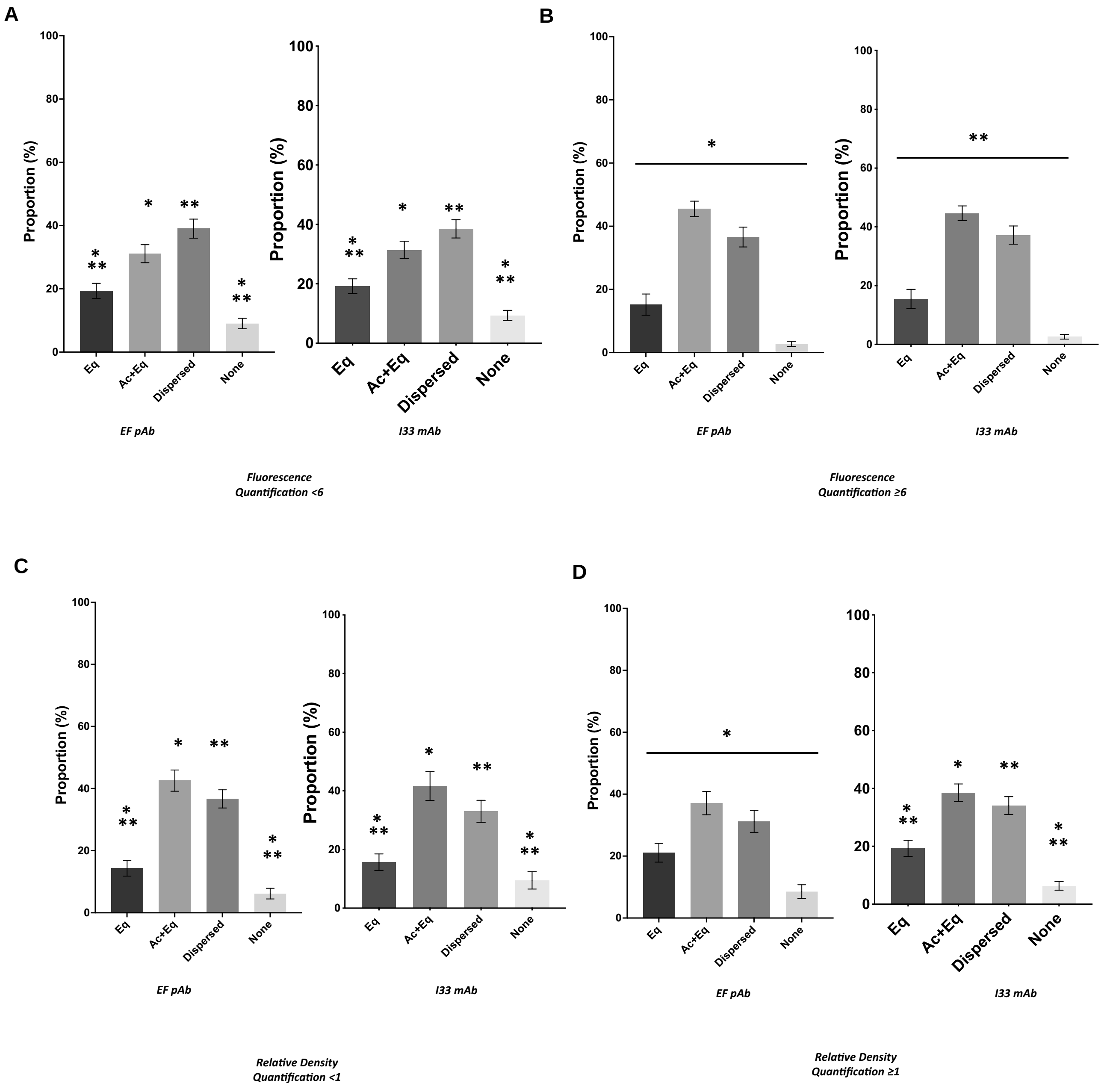

Supplementary Figure S6. Histograms indicating the proportions of sperm exhibiting different PLCζ localization patterns in cases with (A) PLCζ fluorescence cut-off < 6, (B) PLCζ fluorescence cut-off ≥ 6, (C) relative density cut-off < 1, and (D) relative density cut-off ≥ 1. Asterisks (*) indicate a statistically significant ($P \leq 0.05$) difference. Data are indicative of 100 cells (fluorescence quantification) or three repeats (relative density quantification) of sperm examined from 54 patients. a.u, arbitrary units.
