# Supplementary material for: The mammalian sperm factor phospholipase C zeta is critical for early embryo division and pregnancy in humans and mice
Source: Hum Reprod. 2024 Apr 26;39(6):1256–74. doi: 10.1093/humrep/deae078 (PMC11145019; doi:10.1093/humrep/deae078)
Supplement: deae078_Supplementary_Figure_S7 [file deae078_supplementary_figure_s7.pdf]

OXFORD
human reproduction

Human Reproduction, 2024, 1–1
https://doi.org/10.1093/humrep/deae078
Supplementary Data

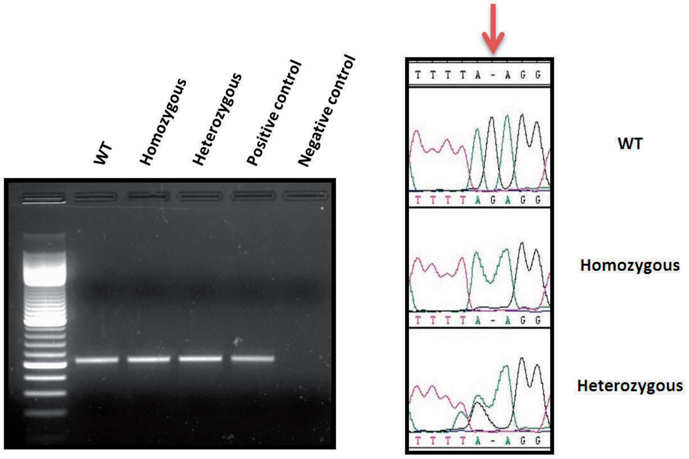

PLCZ Ex3 WT:
ATGGTTTTTGTCAAAGGTTCAGGATGATTTTAGAGGTGGAAAAATCAACGTTGAAATTACTCACAAACTGCTTGAGAAACTTGATTTCCCATGCCACTTTGCTCATGTGAAACATATTTTTAAG

PLCZ Ex3 Mutant:
ATGGTTTTTGTCAAAGGTTCAGGATGATTTTA | AGGTGGAAAAATCAACGTTGAAATTACTCACAAACTGCTTGAGAAACTTGATTTCCCATGCCACTTTGCTCATGTGAAACATATTTTTAAG

Supplementary Figure S7. Genotyping strategy used to confirm presence of desired single base pair deletion used to generate the Exon 3 homozygous strain (E3–/–) strain of mouse. Generated amplicons were submitted for direct sequencing, with homozygous mice exhibiting a deletion of guanine (G).
