# Supplementary material for: The mammalian sperm factor phospholipase C zeta is critical for early embryo division and pregnancy in humans and mice
Source: Hum Reprod. 2024 Apr 26;39(6):1256–74. doi: 10.1093/humrep/deae078 (PMC11145019; doi:10.1093/humrep/deae078)
Supplement: deae078_Supplementary_Figure_S8 [file deae078_supplementary_figure_s8.pdf]

OXFORD
human reproduction

Human Reproduction, 2024, 1–1
https://doi.org/10.1093/humrep/deae078
Supplementary Data

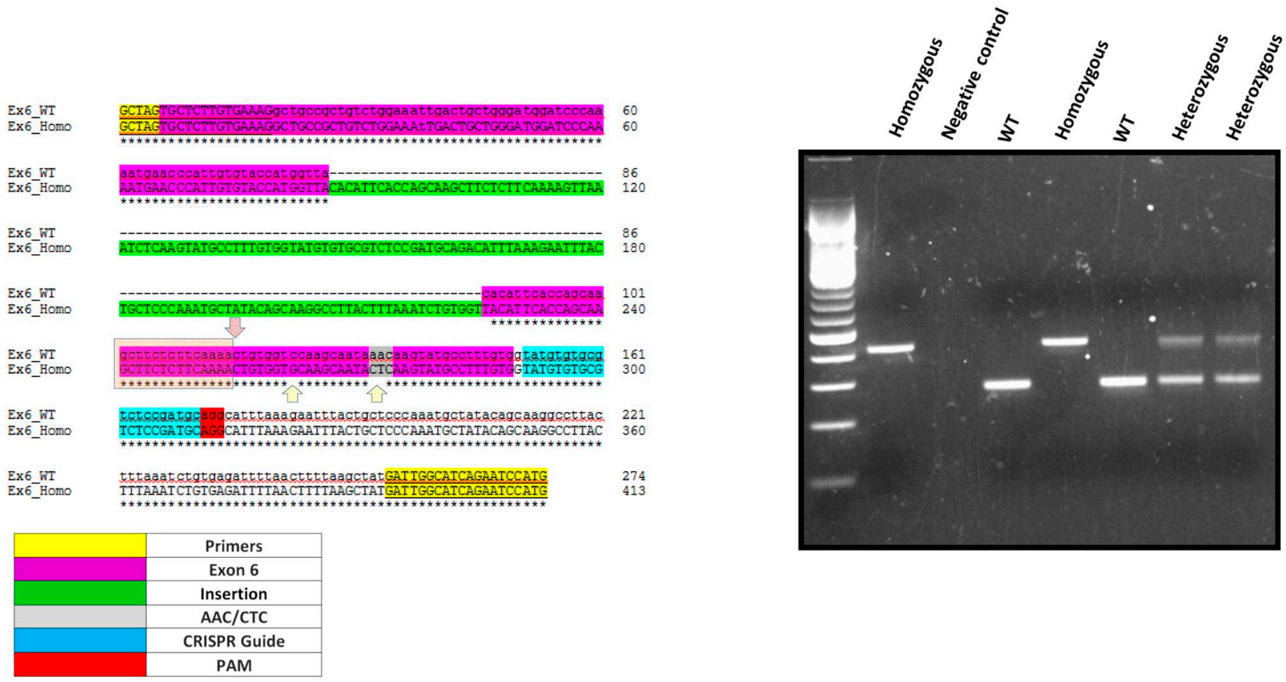

Supplementary Figure S8. Genotyping strategy used to confirm presence of desired single base pair deletion used to generate the Exon 6 homozygous strain (E6−/−) strain of mouse. Generated amplicons for homozygous mice exhibited a significantly higher amplicon compared to WT mice, while heterozygous mice exhibited both amplicons. Direct sequencing confirmed presence of the ×2 inserted ssODN donor sequence, alongside the desired base change.
