# Supplementary material for: The mammalian sperm factor phospholipase C zeta is critical for early embryo division and pregnancy in humans and mice
Source: Hum Reprod. 2024 Apr 26;39(6):1256–74. doi: 10.1093/humrep/deae078 (PMC11145019; doi:10.1093/humrep/deae078)
Supplement: deae078_Supplementary_Figure_S9 [file deae078_supplementary_figure_s9.pdf]

Human Reproduction, 2024, 1–1
https://doi.org/10.1093/humrep/deae078
Supplementary Data

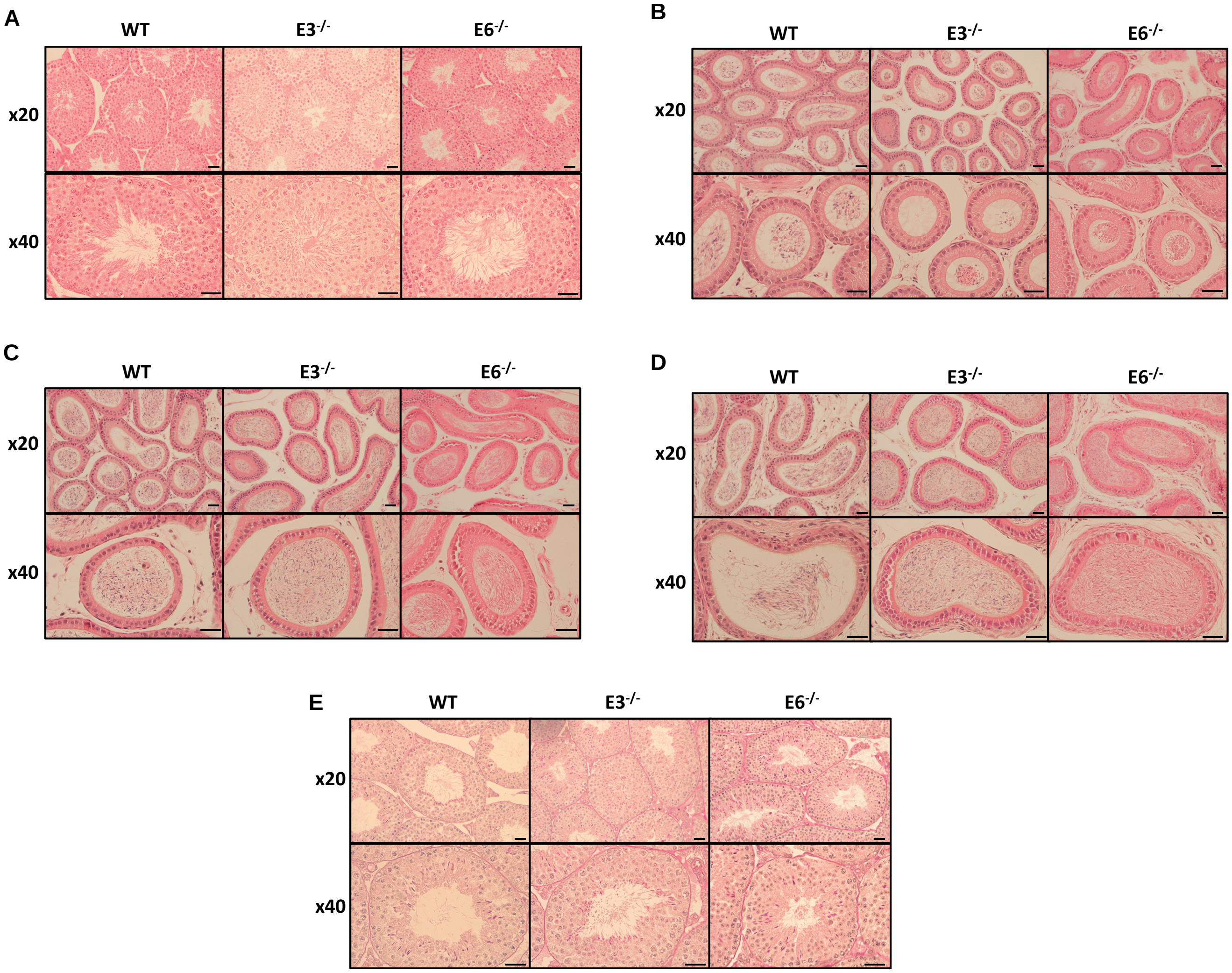

Supplementary Figure S9. Hematoxylin and eosin (H, E) staining of (A) testes and (B) caput, (C) corpus, and (D) cauda epididymides from WT (left-most panels) and PLCζ mutant (E3$^{-/-}$; middle panels; E6$^{-/-}$: right-most panels) adult mice. Sperm integrity was also examined using (**E**) Periodic acid-Schiff (PAS) staining of testes sections from WT and PLCζ mutant mice. Images were taken at 20× (uppermost panels) and at 40× (bottom panels) magnification. Black scale bars indicate 20 μm. Images are representative of three independent repeats.
