# Supplementary material for: The mammalian sperm factor phospholipase C zeta is critical for early embryo division and pregnancy in humans and mice
Source: Hum Reprod. 2024 Apr 26;39(6):1256–74. doi: 10.1093/humrep/deae078 (PMC11145019; doi:10.1093/humrep/deae078)
Supplement: deae078_Supplementary_Figure_S10 [file deae078_supplementary_figure_s10.pdf]

OXFORD
human reproduction
Human Reproduction, 2024, 1–1
https://doi.org/10.1093/humrep/deae078
Supplementary Data

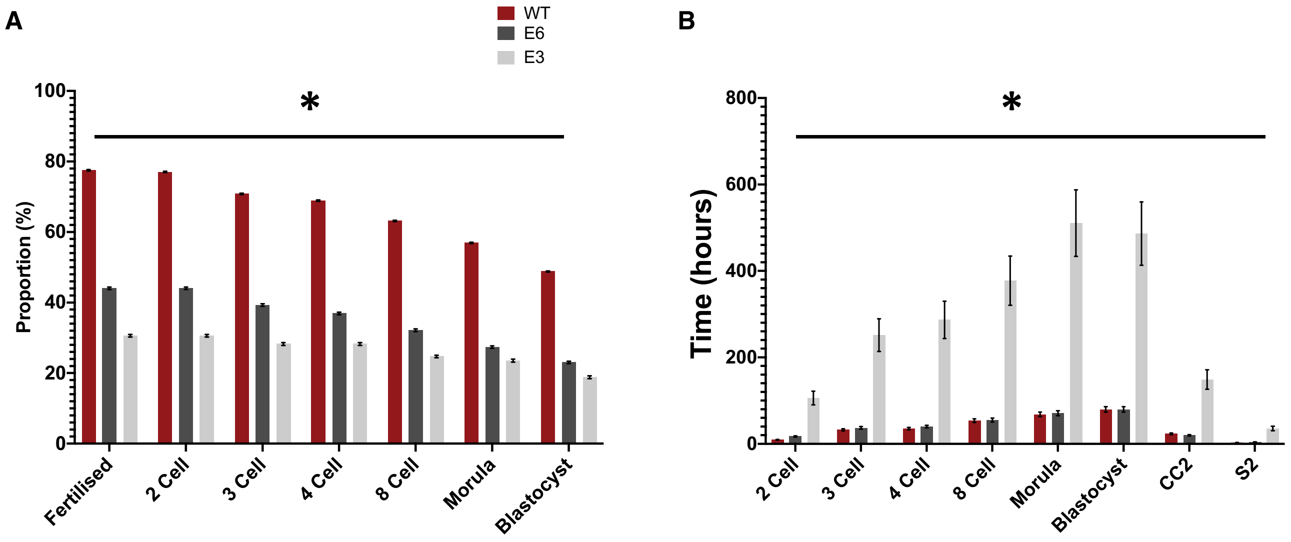

Supplementary Figure S10. Histograms representing embryogenic efficacy of embryos generated following IVF experiments using oocytes from wildtype (WT) females and sperm of WT (red), E3 homozygous (E3−/−; dark grey), and E6 homozygous (E6−/−; light grey) strains of mice (including cases of polyspermy). (**A**) proportions of embryos generated at specific developmental milestones, and (**B**) time taken (h) to reach specific developmental morphokinetic milestones. Asterisk (*) indicates a statistically significant ($P \leq 0.05$) difference, and data are representative of three biological and three technical (3×3) repeats. CC2, time taken for second mitosis (2-cell to 3-cell); S2, time taken for third mitosis (3-cell to 4-cell).
