# Supplementary material for: The mammalian sperm factor phospholipase C zeta is critical for early embryo division and pregnancy in humans and mice
Source: Hum Reprod. 2024 Apr 26;39(6):1256–74. doi: 10.1093/humrep/deae078 (PMC11145019; doi:10.1093/humrep/deae078)
Supplement: deae078_Supplementary_Table_S1 [file deae078_supplementary_table_s1.pdf]

Human Reproduction, 2024, 1–1
https://doi.org/10.1093/humrep/deae078
Supplementary Data

Supplementary Table S1. Oligonucleotide and RNA sequences used in the CRISPR/Cas strategy to generate exon 3 and exon 6 mutant mice.

Oligos	Oligo type	Sequence	Target region
**Non-sense/indel**	crRNA	5′-GUUCAGGAUGAUUUUAGAGGGUUUUA GAGCUAUGCUGUUUUG-3′	Exon 3
**N241L-crRNA**	crRNA	5′-UAUGUGUGCGUCUCCGAUGCGUUUU AGAGCUAUGCUGUUUUG-3′	Exon-6
**ssODN for N241L**	ssODN	5′-ATC CCA AAA TGA ACC CAT TGT GTA CCA TGG TTA CAC ATT CAC CAG CAA GCT TCT CTT CAA AAC TGT GGT GCA AGC AAT ACT CAA GTA TGC CTT TGT GGT ATG TGT GCG TCT CCG ATG CAG ACA TTT AAA GAA TTT ACT GCT CCC AAA TGC TAT ACA GCA AGG CCT TAC TTT AAA TCT GTG AGA TTT TAA CTT TTA AGC TA-3′	Exon-6
**tracrRNA**	tracrRNA	5′-AAACAGCAUAGCAAGUUAAAAUAAGGCUAGUCC GUUAUCAACUUGAAAAAGUGGCACCGAGUCGGUGCU-3′	
