# Supplementary material for: The mammalian sperm factor phospholipase C zeta is critical for early embryo division and pregnancy in humans and mice
Source: Hum Reprod. 2024 Apr 26;39(6):1256–74. doi: 10.1093/humrep/deae078 (PMC11145019; doi:10.1093/humrep/deae078)
Supplement: deae078_Supplementary_Table_S2 [file deae078_supplementary_table_s2.pdf]

OXFORD
human reproduction

Human Reproduction, 2024, 1–1
https://doi.org/10.1093/humrep/deae078
Supplementary Data

Supplementary Table S2. Oligonucleotide sequences used in the genotyping of exon 3 and exon 6 mutant mice.

Primer name	Primer sequence	Application
**Exon 3 Forward**	5′-TTC AGT AAC TCT CAG GCT-3′	Sequencing
**Exon 3 Reverse**	5′-ACT CAA GTA TCT ACT AAT GTC-3′	Sequencing
**Exon 3 Forward**	5′-TTC AGT AAC TCT CAG GCT-3′	Common PCR primer
**Exon 3 Reverse WT**	5′-CGT TGA TTT TTC GAC CTC-3′	PCR primer for WT Plcζ amplicon
**Exon 3 Reverse MU**	5′-CGT TGA TTT TTC CAC CTT-3′	PCR primer for Plcζ exon 3 mutation amplicon
**Exon 3 Reverse CT**	5′-TCG TTG ATT TTT CCA CCT-3′	PCR primer flanking WT or mutated Plcζ exon 3 sequence region as internal control amplification
**Exon 6 Forward**	5′-GCT AGT GCT CTT GTG AAA G-3′	PCR
**Exon 6 Reverse**	5′-CAT GGA TTC TGA TGC CAA TC-3′	PCR
