# Supplementary material for: The mammalian sperm factor phospholipase C zeta is critical for early embryo division and pregnancy in humans and mice
Source: Hum Reprod. 2024 Apr 26;39(6):1256–74. doi: 10.1093/humrep/deae078 (PMC11145019; doi:10.1093/humrep/deae078)
Supplement: deae078_Supplementary_Table_S3 [file deae078_supplementary_table_s3.pdf]

Human Reproduction, 2024, 1–1
https://doi.org/10.1093/humrep/deae078
Supplementary Data

Supplementary Table S3. Oligonucleotide sequences used in sequencing of the top five predicted off-target regions for E3 and E6 mutant mice.

Potential off-target regions	Primer sequence
**E3-1**	F: 5′-GATTTTGGCCGCAGTAGTAGAGG-3′<br>R: 5′-TCCCCTATTCATCATCTTTCAGAGTCAGG-3′
**E3-2**	F: 5′-GACAGCATTCTACATAGCTGTTGTGTTAACC-3′<br>R: 5′-TCTTTCTGGCACTTTTAGACAAATCTGGTTTG-3′
**E3-3**	F: 5′-TTATTTGCCCTTGGCAATTCTCTGC-3′<br>R: 5′-GAGTACACTAGCAACAAAGATGTTAATTTGATGC-3′
**E3-4**	F: 5′-CCCTCCTGATTAACAGCATTGATAGG-3′<br>R: 5′-GTCAAGAAGCCAGAGGCTGG-3′
**E3-5**	F: 5′-TGATGGAGAAGCTCACTTCCAGC-3′<br>R: 5′-GACAGAGACTCCTGAACTCGAATTCTG-3′
**E6-1**	F: 5′-TCGTCGGCAGCGTCTTTGATCAGCGTCCCCTTCC-3′<br>R: 5′-GTCTCGTGGGCTCGGTGAGGTGTTCCACTGAGGGA-3′
**E6-2**	F: 5′-TCGTCGGCAGCGTCTCAGTGACACAAGCCAGAGC-3′<br>R: 5′-GTCTCGTGGGCTCGGGGCTGGCACTCTAGTCACTG-3′
**E6-3**	F: 5′-TCGTCGGCAGCGTCACTTGTATTACCTGCTGCTCC-3′<br>R: 5′-GTCTCGTGGGCTCGGACCCACATGAAAACCACGGA-3′
**E6-4**	F: 5′-TCGTCGGCAGCGTCTGCAGGTTGTGGGTATTACCA-3′<br>R: 5′-GTCTCGTGGGCTCGGTTGGTTTCAAAGCTTTCTCATTAGA-3′
**E6-5**	F: 5′-TCGTCGGCAGCGTCACACCTCTCTCTTCTTGCAGC-3′<br>R: 5′-GTCTCGTGGGCTCGGCCTCCATTCTGGCATGGGTT-3′
