# Supplementary material for: The mammalian sperm factor phospholipase C zeta is critical for early embryo division and pregnancy in humans and mice
Source: Hum Reprod. 2024 Apr 26;39(6):1256–74. doi: 10.1093/humrep/deae078 (PMC11145019; doi:10.1093/humrep/deae078)
Supplement: deae078_Supplementary_Table_S4 [file deae078_supplementary_table_s4.pdf]

OXFORD
human reproduction
Human Reproduction, 2024, 1–2
https://doi.org/10.1093/humrep/deae078
Supplementary Data

Supplementary Table S4. Male patient summary of the 54 cases examined for this study, indicating the sperm concentration (million/ml), semen volume (ml), motility (%), and male/female age (DOB).

Patient number	Count (million/ml)	Volume (ml)	Motility (%)	Morphology	Male DOB	Female DOB	Ovulation protocol	No. of oocytes obtained	Treatment	Fertilization number	Pregnancy	Confirmation type
**1**	126	2.5	78	Normozoospermia	36	40	Long	8	ICSI	6	No	
**2**	11	3	56	Oligoteratozoospermia	35	31	Antagonist	9	ICSI	2	No	
**3**	142	4.5	34	Normozoospermia	38	36	Antagonist	1	IVF	1	No	
**4**	128	1.5	72	Normozoospermia	41	34	Antagonist	19	IVF	6	No	
**5**	12	2.5	35	Asthenoteratozoospermia	34	29	Antagonist	6	ICSI	3	No	
**6**	113	3.5	54	Normozoospermia	35	34	Long	15	ICSI	10	Yes	Biochemical
**7**	14	3	83	Teratozoospermia	32	30	Long	16	½ ICSI and ½ IVF	7	Yes	Ultrasound
**8**	77	2.5	43	Asthenoteratozoospermia	34	38	Antagonist	11	IVF	1	No	
**9**	51	2	20	Teratozoospermia	38	35	Long	15	ICSI	2	No	
**10**	N/A	N/A	N/A	Normozoospermia	N/A	N/A	N/A	N/A	N/A	N/A	N/A	
**11**	27.96	4.5	17	Normozoospermia	32	29	Long	10	ICSI	5	Yes	Ultrasound
**12**	36.29	3	71	Normozoospermia	N/A	37	Long	9	IVF	5	Yes	Ultrasound
**13**	136	5	75	Teratozoospermia	34	34	Antagonist	6	IVF	0	N/A	
**14**	91	3	13	Asthenoteratozoospermia	53	35	Long	31	ICSI	18	N/A	
**15**	137	3	68	Normozoospermia	37	32	Antagonist	11	½ ICSI and ½ IVF	7	No	
**16**	146	3.5	56	Asthenoteratozoospermia	32	33	Antagonist	10	½ ICSI and ½ IVF	3	No	
**17**	121	2	68	Normozoospermia	39	43	Long	8	½ ICSI and ½ IVF	5	No	
**18**	15	2	40	Normozoospermia	34	30	Long	20	½ ICSI and ½ IVF	5	Yes	Ultrasound
**19**	N/A	N/A	N/A	Teratozoospermia	N/A	N/A	Long	24	ICSI	3	Yes	Ultrasound
**20**	2.1	5	23	Oligoasthenozoospermia	41	43	Short	18	ICSI	9	No	
**21**	46	0.7	6	Normozoospermia	49	45	Long	33	ICSI	17	No	
**22**	51	3.5	40	Asthenoteratozoospermia	44	36	Long	18	ICSI	14	No	
**23**	69	1.5	4	Asthenoteratozoospermia	32	29	Long	20	ICSI	9	No	
**24**	N/A	N/A	N/A	N/A	N/A	N/A	Short	15	½ ICSI and ½ IVF	13	No	
**25**	20	4.5	32	Oligoasthenozoospermia	36	34	Long	18	ICSI	6	N/A	
**26**	50	4	65	Teratozoospermia	33	28	Antagonist	19	½ ICSI and ½ IVF	15	N/A	
**27**	107	1.5	76	Normozoospermia	34	32	Long	8	IVF	5	No	
**28**	91	1.5	38	Teratozoospermia	31	31	Long	11	IVF	8	Yes	Ultrasound
**29**	66	2	3	Asthenoteratozoospermia	34	31	Long	12	ICSI	7	Yes	Ultrasound
**30**	153	6.5	16	Normozoospermia	40	36	Long	17	IVF	1	Yes	Biochemical
**31**	37	4	65	Teratozoospermia	35	31	Long	19	½ ICSI and ½ IVF	13	No	
**32**	35	1	17	Asthenoteratozoospermia	43	42	Long	9	ICSI	3	No	
**33**	13	2.5	29	OAT	37	32	Antagonist	18	ICSI	8	No	
**34**	N/A	N/A	N/A	N/A	N/A	N/A	N/A	N/A	N/A	N/A	N/A	
**35**	47.41	4	38	Teratozoospermia	37	35	Long	18	ICSI	10	N/A	
**36**	9.7	3	28	OAT	37	35	Long	48	ICSI	25	N/A	
**37**	168	3.5	69	Normozoospermia	31	31	Long	8	IVF	4	No	
**38**	139	2.5	61	Normozoospermia	32	28	Long	11	ICSI	5	Yes	Ultrasound
**39**	7.7	6	8	Asthenozoospermia	N/A	29	Antagonist	32	ICSI	24	N/A	
**40**	143	1	27	Asthenoteratozoospermia	32	34	Antagonist	12	ICSI	3	No	
**41**	13.1	4.5	42	Asthenoteratozoospermia	30	28	Antagonist	16	ICSI	10	No	
**42**	145	4	43	Teratozoospermia	32	28	Antagonist	0	ICSI	0	N/A	
**43**	7.8	5	50	OAT	36	27	Long	4	ICSI	2	No	
**44**	14	5	9	Asthenoteratozoospermia	32	26	Long	17	ICSI	8	Yes	Ultrasound
**45**	336	7	68	Normozoospermia	33	34	Long	9	PGD	6	No	
**46**	12.6	3	38	Teratozoospermia	36	30	Antagonist	34	½ ICSI and ½ IVF	10	N/A	
**47**	65.3	2.5	38	Teratozoospermia	45	39	Antagonist	10	IVF	7	No	
**48**	16	1.5	39	Teratozoospermia	35	33	Long	9	ICSI	3	No	

(continued)

Supplementary Table S4. Continued

Patient number	Count (million/ml)	Volume (ml)	Motility (%)	Morphology	Male DOB	Female DOB	Ovulation protocol	No. of oocytes obtained	Treatment	Fertilization number	Pregnancy	Confirmation type
**49**	199	1.5	60	Teratozoospermia	30	31	Long	19	ICSI	11	Yes	Ultrasound
**50**	26.6	2	71	Oligoteratozoospermia	37	35	Antagonist	13	ICSI	8	No	
**51**	60	1.5	35	Teratozoospermia	37	34	Long	20	ICSI	9	No	
**52**	97	2	46	Teratozoospermia	30	24	Antagonist	33	ICSI	6	N/A	
**53**	159	4	71	Teratozoospermia	40	33	Antagonist	34	IVF	24	N/A	
**54**	77	2	62	Normozoospermia	39	43	Long	10	ICSI	8	No	

Where possible, morphological indications are also listed. N/A, not available; OAT, oligoasthenozoospermia.
