# Supplementary material for: The mammalian sperm factor phospholipase C zeta is critical for early embryo division and pregnancy in humans and mice
Source: Hum Reprod. 2024 Apr 26;39(6):1256–74. doi: 10.1093/humrep/deae078 (PMC11145019; doi:10.1093/humrep/deae078)
Supplement: deae078_Supplementary_Table_S5 [file deae078_supplementary_table_s5.pdf]

Human Reproduction, 2024, 1–1
https://doi.org/10.1093/humrep/deae078
Supplementary Data

Supplementary Table S5. Correlative analysis between morphokinetic milestones and male/female age, and proportions of successful pregnancy from corresponding human patients undergoing fertility treatment.

	Motility (%)	Count (million/ml)	Volume (ml)	Male Age (Years)	tPNa		tPNf	CC2	S2	t2	t3	t4	t5	t6	t7	t8	KIDScore <4	KIDScore ≥4
**Male age (years)**	**$r = -0.3$ ($P < 0.05$)**	n.s.	n.s.	n.s.	n.s.	n.s.	n.s.	n.s.	n.s.	n.s.	n.s.	n.s.	n.s.	n.s.	n.s.	n.s.	n.s.	n.s.
**Female age (years)**	n.s.	n.s.	n.s.	n.s.	n.s.	n.s.	n.s.	n.s.	n.s.	n.s.	n.s.	n.s.	n.s.	n.s.	n.s.	n.s.	n.s.	n.s.
**Pregnancy (%)**	n.s.	n.s.	n.s.	n.s.	n.s.	n.s.	n.s.	n.s.	n.s.	n.s.	n.s.	n.s.	n.s.	n.s.	n.s.	n.s.	n.s.	n.s.

Statistically significant ($P \leq 0.05$) differences are indicated, along with the corresponding Pearson's correlation coefficient (r; positive values indicate a positive correlation). n.s., non-significant differences.

© The Author(s) 2024. Published by Oxford University Press on behalf of European Society of Human Reproduction and Embryology.
Human Reproduction, 2024, 00, 1–35
https://doi.org/10.1093/humrep/deae078
Original Article
